# Cell therapy efficacy and safety in treating tendon disorders: a systemic review of clinical studies

**DOI:** 10.1186/s40634-022-00520-9

**Published:** 2022-08-30

**Authors:** Seyed Peyman Mirghaderi, Zahra Valizadeh, Kimia Shadman, Thibault Lafosse, Leila Oryadi-Zanjani, Mir Saeed Yekaninejad, Mohammad Hossein Nabian

**Affiliations:** 1 Center of Orthopedic Trans-Disciplinary Applied Research (COTAR), Tehran University of Medical Sciences, Tehran, Iran; 2 Students’ Scientific Research Center (SSRC), Tehran University of Medical Sciences, Tehran, Iran; 3 Alps Surgery Institute: Hand, Upper Limb, Brachial Plexus, and Microsurgery Unit (PBMA), Clinique Générale d’Annecy, Annecy, France; 4 Department of Orthopedic and Trauma Surgery, Shariati Hospital and School of Medicine, Tehran University of Medical Sciences, Tehran, Iran; 5 Department of Epidemiology and Biostatistics, School of Public Health, Tehran University of Medical Sciences, Tehran, Iran

**Keywords:** Cell therapy, Regenerative medicine, Safety, Tendinopathy, Tendons, Stem cells

## Abstract

**Purpose:**

Despite substantial animal evidence, cell therapy in humans remains in its infancy. The purpose of this study was to examine the potential therapeutic effects and safety of cell therapy in the treatment of tendon disorders.

**Methods:**

According to the PRISMA guideline, a systematic review was performed on clinical studies concerning cell therapy in tendon disorders. A comprehensive search including the 5 databases of MEDLINE, Embase, Scopus, Web of Science, and Cochrane Library until December 2021 was carried out and associated with hand searching. The quality of the eligible studies was assessed using the tools suggested by Cochrane recommendations. Qualitative synthesis was performed in 2 tables and discussed separately for rotator cuff, elbow, patella, Achilles, and gluteal tendons.

**Results:**

Through 6017 records, 22 studies were included in the qualitative synthesis, including 658 patients. All the studies administered autologous cells, except one that used allogenic adipose-derived mesenchymal stem cells (Allogenic AD-MSC). Almost all studies demonstrated the safety of cell injection in their follow-up period with no serious side effects or immunologic reactions, with only a few related minor adverse events in some cases.

The included studies showed the effectiveness of cell injection in tendinopathies of different sites, rotator cuff, elbow, patella, Achilles, and gluteal tendons. Among the rotator cuff studies, 4 comparative studies claimed that cell therapy is a more efficient treatment with a lower retear rate and pain level compared to the control group. However, one study found no differences between the groups. No controlled study has been performed on elbow tendinopathies, but 5 case series demonstrated the effectiveness of cell injection in elbow tendon disorders. For Achilles tendinopathies, only one randomized controlled trial (RCT) found that both cell therapy and control groups showed significant pain reduction and functional improvement with no statistical differences at the 6 months follow-up, but the cell therapy group had improved faster at earlier follow-ups. Patellar tendinopathy was studied in 2 RCTs, one did not show a significant difference and the other showed superior improvement compared to controls.

**Conclusion:**

Cell therapy showed promising results and the available evidence suggests that it is safe at several sites of tendon disease. Based on available evidence, cell therapy should be suggested in specific conditions at each site. To approve cell therapy for tendon diseases, randomized clinical trials are required with a large sample size and long-term follow-ups.

**Level of evidence:**

IV

**Supplementary Information:**

The online version contains supplementary material available at 10.1186/s40634-022-00520-9.

## Introduction

As widespread and chronic disorders, tendinopathies can cause severe economic, social, physical, and psychological burden for patients [1, 2]. It is estimated that lower limb tendinopathy occurs at an incidence of 11.83 and a prevalence of 10.52 per 1000 person-years, respectively. This number increased in the sporting population up to 14.4% and in elite volleyball players up to 45% [1]. Due to insufficient blood supply, tendon tissue cannot efficiently repair its defect and reform [3]. Moreover, the tendon tends to form fibrous tissue and scars in the healing process, thus leading to adhesion formation [4]. Therefore, tendinopathy and tendon rupture impairs the patient’s ability and function [3]. Thus, the treatment of tendon disorders is a significant challenge for orthopaedic surgeons [4]. Various treatments, both operative and non-operative, for the restoration of tendon function have been discussed [5, 6]. Currently, operative repair is the treatment of choice after the failure of conservative treatments [5].

Cell therapy, performed through prepared cells injection, shows encouraging results [7–10]. The most common cells used in this method are mesenchymal stem cells (MSCs), multipotent stem cells primarily found in bone marrow and capable of differentiating into bone, tendon, cartilage, muscle, ligament, fat, and marrow stroma. MSCs can be applied to the injury site or delivered on a scaffold [1, 11]. Other cells can be used in tendinopathy such as human embryonic stem cells (hESCs), bone marrow cells, bone marrow mononuclear cells (BMMCs), and adipose-derived stem cells (ASCs) [2]. The rationale behind cell therapy for tendon disorders is that fibroblastic cells derive from undifferentiated MSCs. Cells of this type are responsible for tendon healing through synthesizing collagen after tendon damage [3, 4, 12].

The results of previous studies using cell therapy for tendon healing were promising, and cell injections such as MSC demonstrated a significant pooled effect size for pain and functional scores, as well as structural healing in radiologic and arthroscopic investigations [13–17]. Some studies have presented superior radiological and clinical outcomes for cell therapy in tendon disease [14], while others have claimed faster healing regardless of similar outcomes in the end [15]. Although previous studies have supported cell therapy, some have encountered serious limitations such as non-randomized allocation and unavoidable selection biases, low quality of the method, heterogeneity, disagreement over the details of the method, and short-term follow-ups [9]. A recent meta-analysis reviewed 4 prospective studies, suggesting the high efficacy of MSC therapy in tendon disorder and its promising outcome in respect to radiologic, arthroscopic, and functional parameters [18]. In addition, a systematic review of stem cell therapy identified 8 trials with significant bias, and thus, they could not conclude that it is safe [9].

Regardless of massive animal evidence, cell therapy in humans is in its infancy. This systematic review provides a summary of the current findings on the potential therapeutic effect of cell therapy and its safety in healing tendon disorders. This study sought to compare the beneficial effect of cell therapy based on the injury site, and the type of cells injected and their source.

## Materials and methods

### Search strategy and screening

The study was performed according to the Preferred Reporting Items for Systematic Reviews and Meta-Analyses (PRISMA) guidelines (see Additional file 1) [19]. A protocol for this review has been registered in the International Prospective Register of Systematic Reviews (PROSPERO); registration ID: CRD42021251539; https://www.crd.york.ac.uk/PROSPERO).

The 5 databases of MEDLINE/PubMed, EMBASE, SCOPUS, Web of Science, and Cochrane Library were searched for clinical studies that applied cell therapy for tendon disease treatment from inception until March 22, 2021. We updated our search on December 26, 2021. Our search strategy consisted of numerous keywords and database-specific subject heading vocabulary to identify studies regarding cell therapy for tendon disease, which includes these concepts without any prior restriction: “Cell therapy” AND “Tissue Engineering” OR “Regenerative Medicine” AND “Tendon.” The thorough search strategy is available in the supplementary material (Tables S1, S2, and S3, (see Additional file 2)). The search query was changed to some extent based on the search rules of each database. Citation searching and forward citation screening were performed on the potentially eligible articles, and a reference was included when it met our eligibility criteria.

All records were imported to the Covidence online systematic review software (https://www.covidence.org). After removing duplicates, 2 reviewers (S.P.M and Z.V) separately screened all the imported articles to find eligible studies based on the distinct inclusion/exclusion criteria. A study was included if it had gained 2 “yes” votes in both steps of title/abstract screening and consequent full-text screening. For full-text papers not included in the analysis, the reasons for exclusion were documented. Here and in the following sections, any conflicts were resolved through discussion and consulting with the corresponding author (M.H.N).

### Inclusion and exclusion criteria

All original studies that had administered any type of cells to treat patients with tendon disease were included in the review. There was no restriction on the route through which cells were administered. The included studies had to represent the patient-reported outcome measures (PROMs) or paraclinical imaging investigations as to their primary outcome. The exclusion criteria included lack of administration of cells as intervention, non-English articles, non-human studies (animal or in vitro studies), patients with another major interfering morbidity, case reports, reviews, congress abstracts, commentaries, and book chapters, and lack of availability of the full-text (after attempts to receive the text from the authors or the journal via Email). No other restrictions were applied to the inclusion of studies.

### Assessment of study quality

Again the same 2 reviewers independently evaluated the selected studies in terms of their quality and risk of bias. The Cochrane Risk of Bias tool V 2.0. (RoB 2) [20] was used for randomized controlled trials (RCTs). This tool assesses the study based on the 6 subsets of selection bias, performance bias, detection bias, attrition bias, reporting bias, and other potential biases [20]. According to the Cochrane recommendations, studies were rated in each topic as high, low, or unclear. Finally, the overall estimation of the study quality was expressed as high risk, low risk, or some concern.

To evaluate the quality of non-RCTs, here we assumed clinical trials that groups were not divided randomly, the Joanna Briggs Institute (JBI) Critical Appraisal Checklist for Quasi-experimental studies [21] was used. The National Institutes of Health (NIH) quality assessment tool for case-series studies was used for the case studies without a control group [22]. This tool evaluates the quality of case studies based on selection, comparability, and description of the population, intervention, result, and statistical method. Both NIH and JBI tools described appraising the articles with 9 questions. Each question received a score of 1 (yes) or 0 (no). Greater scores are considered as high quality. A score of ≥8, 5-7, and ≤ 4 was associated with high, moderate, and low quality.

### Levels of evidence

The level of evidence was written as declared in the original study. The Centre for Evidence-Based Medicine (CEBM) guideline in Oxford, UK, was used to detect the level of evidence of studies that had not provided it [23].

### Data collection and abstraction

Data was extracted from the selected studies and entered into a pre-designed table for evidence synthesis by 2 reviewers (S.P.M and Z.V) independently. The characteristics and conclusions are presented in one table, and the study’s raw results in another. The extracted information included the first author’s family name, year of publication, country, type of study design, study groups and their population, sex, age, type and site of injury, size of the lesion, injected cell type and source, follow-up duration, number of cells injected and route, surgery procedure, outcome measure, results and outcomes, and the study’s conclusion and level of evidence. For the studies that had not reported their results as numbers in a table, reviewers extracted the approximate values from the figures and diagrams or emailed the corresponding author to receive the data. Furthermore, any adverse events related to cell injection, including abnormal signs, symptoms, or diseases, were extracted.

### Data synthesis and statistical analysis

Kappa (κ) values were used to assess the inter-reviewer agreement for article screening. Based on a priori category classification, substantial agreement was κ > 0.60, moderate agreement 0.21 < κ < 0.60, and slight agreement κ < 0.21 [24]. Descriptive statistics (e.g., means, ranges, and variance measures) are presented where applicable. A meta-analysis was not performed due to the small number of studies with similar outcome measures and high heterogeneity.

## Results

### Search results

**Fig. 1:**
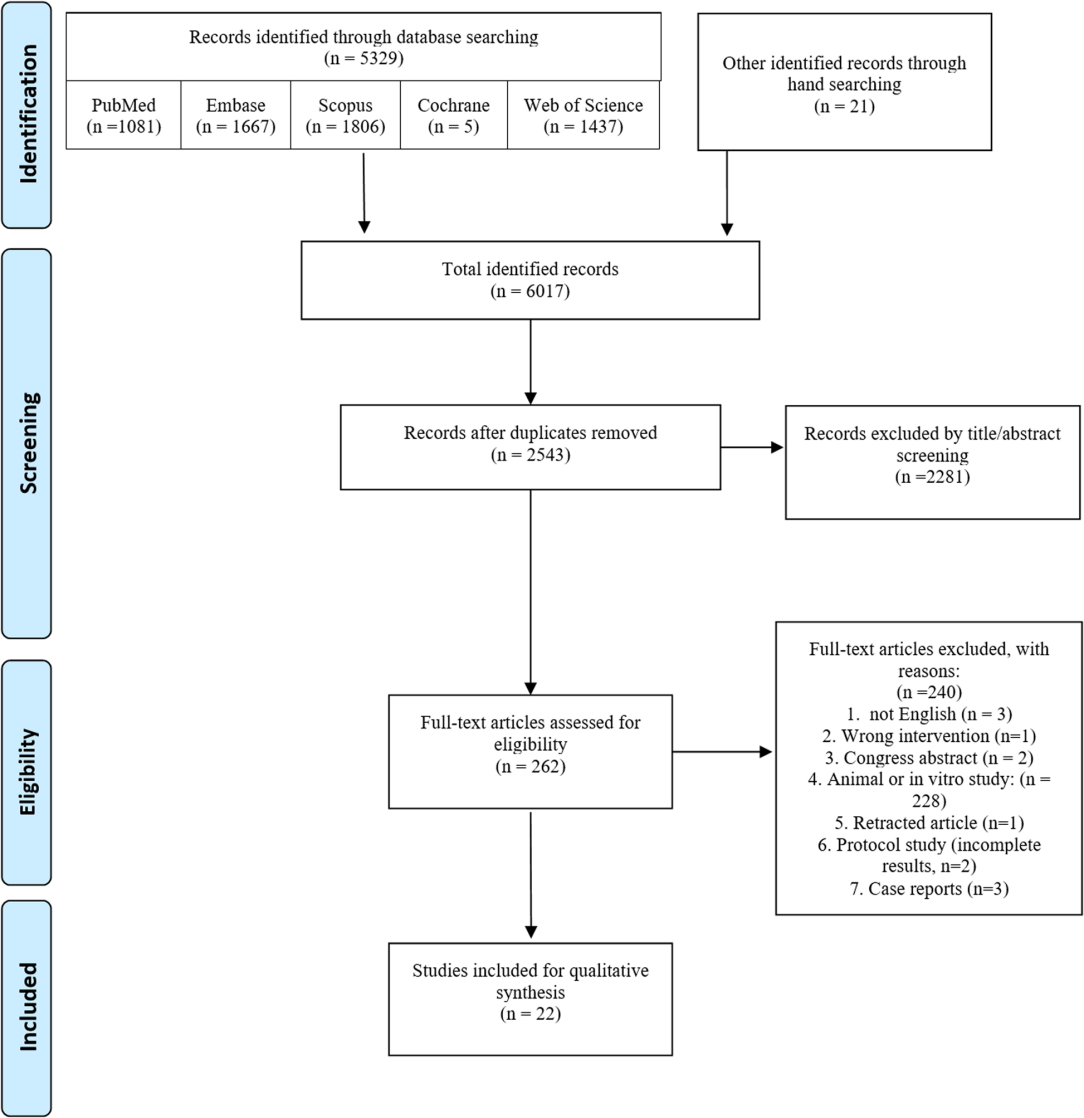
Prisma flow diagram of the study’s selection process

A total of 6017 records were retrieved from the search and hand searching (Fig. 1). After removing duplicates, 2543 studies were screened by title and abstracts. Subsequently, the remaining 262 articles were evaluated by full-text for eligibility, and 22 were selected [14, 15, 25–44]. In terms of the title and abstract screening (κ = 0.89; 95% CI:, 0.86 - 0.92), as well as the full text (κ = 0.85; 95% CI,: 0.81 - 0.89) there was excellent agreement across reviewers. Three relevant case reports were excluded; their results are presented in the supplementary material (table S6, (see Additional file 2)) [45–47]. Moreover, 2 articles were excluded [48, 49] because a new update with a longer follow-up was published [30, 44]. A technical note was excluded because of the lack of follow-up outcomes [18]. The details of the study selection process are illustrated in Fig. 1.

### Included studies

The selected studies consist of 658 patients cumulatively, were published between 2009 and 2021, and were performed in the USA (*n* = 6), South Korea (*n* = 5), UK (*n* = 2), Brazil (*n* = 2), Spain (*n* = 2), Italy (*n* = 1), France (*n* = 1), Australia (*n* = 1), Argentina (*n* = 1), and India (*n* = 1). The studies consist of 13 case series (interventional study without control group), 6 RCTs, and 3 non-RCTs (clinical trials with non-randomized control groups). The characteristics of the studies are presented in Table 1 and divided based on the site of the injury (rotator cuff: 10 studies; elbow: 5 studies; Achilles: 3 studies; patellar: 3 studies; gluteal: 1 study) (Table 1).

**Table 1:** Included studies characteristics

ID	Study (first author, year)	Study design	Type and Site of injury	Groups	Population	Cell type	Cell source	Follow up	Surgical techniques	Conclusion	Level of evidence
**Rotator cuff**											
1	Ellera Gomes 2011	Case-series	Complete rotator cuff tear (Open surgical repair + injection BMMC)	BMMC group (*N* = 14) no control group	14	Autologous BMMC	BMMC (from the posterior iliac crest)	12 months	Open surgical repair	BMMC implantation in a patient with rotator cuff sutures is safe and has promising results compared to historical data (patients underwent surgical procedures without stem cells application).	4
2	Hernigou P 2014	Non-RCT	Rotator cuff tear with size of 1.5-3.5 cm (Arthroscopic surgical repair + MSCs)	1. MSC group (N = 45) 2. Control group (*N* = 45)	90 (45 each groups)	Autologous MSC	BMC (from anterior iliac crest bone)	10 years<	Arthroscopic surgical repair	More prominent and earlier (2 months earlier, *p* = 0.04) healing in MSC-treatment group, total healing more likely achieved when MSCs> 2500 cell.mL^−1^	3
3	C. J. Centeno 2015	Case series	Partial or full-thickness rotator cuff tear (< 1.5 cm)	BMC + PRP + PL (*N* = 102 patients with 115 involved shoulders which ***N*** **= 81** arm with rotator cuff tear)	81	Autologous MSC	BMC (from posterior superior iliac crest)	11.2 ± 10 months	No surgery	Rotator cuff tear patients treated with BMC showed significant pain and functional improvement	4
4	Kim, Y. S 2017	Non-RCT	full-thickness rotator cuff tear (AD-MSC + Arthroscopic surgical repair)	1. Arthroscopic rotator cuff repair alone group (N = 35) 2. AD-MSC group + arthroscopic repair (*N* = 35)	70 (35 each groups)	Autologous AD-MSC	Buttock fat pad	28.3 ± 3.8 months	Arthroscopic rotator cuff repair	AD-MSC injection along with rotator cuff repair reduce retear rate significantly, but no clinical differences at the end of the follow up comparing to controls	3
5	S. J. Kim 2017	Case-series	Partial rotator cuff tear	BMC + PRP group (N = 12) No control group	12	Autologous MSC	BMC (from anterior iliac crest bone)	3 months	No surgery	Injection of BMC–PRP to the rupture site leads to improvement of the reduction of the clinical symptoms of tear size.	4
6	S. J. Kim 2018	Non-RCT	Partial tear of the rotator cuff tendon	1. BMC + PRP group (N = 12) 2. Physical therapy (*N* = 12)	24 (12 each groups)	Autologous MSC	BMC (from anterior iliac crest bone)	3 months	No surgery	BMC-PRP injection improve VAS and ASES scores (improve pain and shoulder function), tear size changes, and MMT did not statistically differ among groups	3
7	J. R. Lamas 2019 (It was stopped due to adverse effects in both groups.)	RCT	Full-thickness rotator cuff tear	1. BM-MSCs + type I collagen membrane (OrthADAPT™) (N = 8) 2. Only type I collagen membrane (*N* = 5)	13 (8 intervention group and 5 controls)	Autologous BM-MSC	BMC (right posterior superior iliac spine)	12 months	Arthroscopic rotator cuff repair	The authors of the study decided to terminate the study prematurely because four patients experienced postoperative complications. A re-rupture was observed in 3/5 control subjects (60%) and in 5/8 treatment subjects (62.5%). The complications experienced by both study groups could not be related to the autologous MSCs but to the scaffold (OrthADAPTTM). However, the Constant score was significantly higher in the treatment group (*p* = 0.007).	1
8	Hurd, J. L 2020	RCT	Partial-thickness rotator cuff tears	1. UA-ADRCs (*N* = 11) 2. Methylprednisolone (N = 5)	16 (11 intervention group and 5 controls)	unmodified, autologous adipose-derived regenerative cells (UA-ADRCs)	Periumbilical abdominal area, bilateral flanks, or medial thigh	13 months	No surgery	UA-ADRCs application in sPTRCT patients is safe and significantly improved function of the shoulder compared to the control group, with no side effects.	1
9	C. H. Jo 2020	Case series	Partial-thickness rotator cuff tear	1. AD-MSC Low dose (N = 3) 2. AD-MSC Mid dose (*N* = 3) 3. AD-MSC High dose (*N* = 13) With no control group	19	Autologous AD-MSCs	Abdominal subcutaneous fat	24 months	No surgery	Intratendinous injection of AD-MSCs is a safe and effective treatment for partial tears of rotor cuff	3
10	L. N. Muench 2020	Case series	Rotator cuff tear (Arthroscopic rotator cuff repair)	Arthroscopic rotator cuff repair + BMC + PRP + subacromial bursa (*N* = 16)	16	Autologous BMC	BMC (from proximal humeral head)	12.6 ± 1.8 (12<) months	Arthroscopic rotator cuff repair	This study showed that arthroscopic rotator cuff repair augmented with BMC improves patient function	4
**Elbow**											
11	D. Connell 2009	Case series	Refractory lateral epicondylitis (tennis elbow)	Elbow CEO tendinosis group (N = 12) without control group	12	Autologous tenocyte-like cell (derived from dermal fibroblast)	Skin	6 months	No surgery	Tendon like cells have a therapeutic effect on refractory CEO tendinosis	4
12	A. Singh 2014	Case series	Previously untreated Lateral epicondylosis (tennis elbow)	Elbow CEO tendinosis BMC injected group (*N* = 26) without control group		Autologous MSC	BMC (from anterior-superior iliac spine)	3 months	No surgery	significant improvement of pain relief and recovery from the disease following a single injection of BMC	4
13	A. Wang 2015	Case series	Severe refractory Lateral Epicondylitis (tennis elbow)	Tenocytes injection (*N* = 15)	15	Autologous Tenocytes	Patellar tendon	4.5 years (range, 3.1-5.2)	No surgery	Tenocyte injection for Lateral Epicondylitis treatment showed acceptable results in function and structure improvement.	4
14	Lee, S. Y 2015	Case series	Chronic and intractable Lateral epicondylosis (tennis elbow)	1. AD-MSC low dose (N = 6) 2. AD-MSC high dose (N = 6) no control group	12 (6 each groups)	**Allogenic** AD-MSC	Human subcutaneous fat tissue	13 months	No surgery	Allogenic AD-MSC injection for lateral epicondylosis treatment is safe and effective.	4
15	M. Khoury 2021	Case series	Chronic, recalcitrant lateral elbow tendinopathy (LET)	AD-MSC (*N* = 18) no control group	18	Autologous AD-MSC	Periumbilical zone	6 months	No surgery	Recalcitrant LET in tennis players showed clinical improvement and anatomical repair after autologous ASCs injection.	
**Achilles**											
16	K. Tate-Oliver 2013	Case- series	Achilles tendinosis and interstitial tears (N = 3)	1. HD-PRP + AD-tSVF (N = 2) 2. HD-PRP + AD-tSVF + BMC (N = 1)	3	Autologous adipose graft (AD-tSVF) plus additive of HD-PRP	Lower abdomen-flank area (male and female) or lateral thigh-buttock area (females)	3-4 years	No surgery	Use of AD-SVF and HD-PRP and/or BMAC is safe and a good option for Achilles tendonitis management without surgery	4
17	Stein, B. E 2015	Case-series	Sport-related Achilles tendon tear (open repair + BMC)	BMC injection group (*N* = 27) No control group	27	Autologous MSC	BMC	29.7 ± 6.1 months	open repair	Patient with Achilles tendon repairs treated with BMAC injection shows a great functional rate of return to sport, rehabilitation progress, and single-limb heel raise outcomes.	4
18	Usuelli, F. G 2017	RCT	Recalcitrant non-insertional Achilles tendinopathy	1. PRP group (*N* = 23) 2. SVF group (*N* = 21)	44 (21 intervention group and 23controls)	Autologous Adipose-derived SVF	Subcutaneous adipose tissue	6 months	No surgery	We can use both PRP and SVF to treat recalcitrant Achilles tendinopathy, and it’s safe and effective. However, we can obtain results faster in SVF treatment.	1
**Patellar**											
19	A. W. Clarke 2011	RCT	Refractory patellar tendinosis	**Patellar tendinopathy (*N*** **= 60, in 46 patients)** 1. Plasma-only injection group (N = 27) 2. Tenocyte-like Cell + plasma injection group (*N* = 33)	60 knees (46 patients, 33 in the intervention group)	Autologous skin-Derived Tenocyte-like Cells	Skin	6 months	No surgery	Patellar tendinopathy treated with skin-derived tendon-like cells can be safely treated in the short term, with a significantly better outcome than that achieved with plasma alone.	1
20	C. Pascual-Garrido 2012	Case series	Chronic patellar tendinopathy	BM-MNC group (N = 8) No control group	8	Autologous BM-MNC	BMC (from anterior iliac crest bone)	5 years (range, 3–6)	No surgery	BM-MNC therapy improves chronic patellar tendinopathy after nonoperative treatment’s failure	4
21	G. Rodas 2021	RCT	Initial, unilateral, chronic patellar tendinopathy with an intratendinous lesion > 0.3 mm	1. Lp-PRP group (N = 10) 2. MSC group (N = 10)	20 (10 each groups)	Autologous BM-MSC	Bone marrow	12 months	No surgery	This study confirmed that treatment with BM-MSC or Lp-PRP could reduce the pain; however, BM-MSC is more effective	2
**Gluteal**											
22	D. A. V. Rosário 2021	RCT	Gluteal tendinopathy	1. Corticosteroid group (*N* = 25) 2. BMC group (N = 15)	40 (15 intervention group and 25 controls)	Autologous MSC	BMC	6 months	No surgery	This study approve that BMAC is safe and effective to treat gluteal tendinopathy	2

*Abbreviations*: *BMC* Bone marrow concentrate, *PRP* Platelet-rich plasma, *VAS* Visual analog scale, *MMT* Manual muscle test, *ASES* American Shoulder and Elbow Surgeons, *NSAID* Non-steroidal anti-inflammatory drugs, *BM-MNC* Bone marrow mononuclear cell, *IKDC* International knee documentation committee, *KOOS* Knee injury ad osteoarthritis outcome score, *SF12* Short Form-12, *ADL* Activities of daily living, *QOL* Quality of life, *AD-MSC* Adipose-derived MSC, *EGF* Epidermal growth factor, *SPADI* Safety and the shoulder pain and disability index, *NCI – CTCAE v4.0* National Cancer Institute - Common Terminology Criteria for Adverse Events version 4.0 scale, *AE* Adverse event, *PRTEE* Patient-Rated Tennis Elbow Evaluation, *IQR* Interquartile range, *RCT* Randomized controlled trial, *CEO* Common extensor origin, *KSS* Knee Society Score, *OA* Osteoarthritis, *PL* Platelet lysate, *DASH* Disabilities of the arm, shoulder and hand, *NPS* Numeric pain scale, *MCID* Minimal clinically important difference, *UCLA* University of California, Los Angeles, *MEPI* Modified Mayo clinic performance index for the elbow, *TEAEs* Treatment emergent adverse events, *VISA* Victorian Institute of Sport Assessment, *sPTRCT* Symptomatic, partial-thickness rotator cuff tears, *UA-ADRC* Uncultured, unmodified, autologous adipose-derived regenerative cell, *SVF* Stromal vascular fraction, *BMMC* Bone marrow mononuclear cells, *US* Ultrasound, *HD-PRP* High-density platelet rich plasma, *AD-tSVF* Autologous adipose-derived tissue stromal vascular fraction, *Lp-PRP* Leukocyte-Poor Platelet-Rich Plasma, *SANE* Single Assessment Numerical Evaluation, *UA-ADRCs* Unmodified, autologous adipose-derived regenerative cells, *UEFS* Upper Extremity Functional Scale

### Methodological quality

The overall quality assessment of the included studies is presented in Table 2; 15 studies have high methodological quality (68.2%), 4 of them have moderate quality or some concern exists regarding biases (18.2%), and 3 studies seemed to have low methodological quality (13.6%).

**Table 2:** Quality assessment of included studies

	Study	Tool	Questions									Overall quality	
	**Case series and non-RCTs**		1	2	3	4	5	6	7	8	9	Assessment	**Score**
1	Hernigou P	JBI	Yes	Yes	Yes	Yes	Yes	Yes	Yes	Yes	Yes	High	9
2	S. J. Kim	NIH	Yes	Yes	No	NR	Yes	Yes	No	Yes	Yes	Moderate	6
3	S. J. Kim	JBI	Yes	Yes	Yes	Yes	Yes	Yes	Yes	Yes	Yes	High	9
4	C. Pascual-Garrido	NIH	Yes	Yes	Yes	NR	Yes	Yes	Yes	Yes	Yes	High	8
5	D. Connell 2009	NIH	Yes	No	Yes	CD	Yes	Yes	No	Yes	Yes	Moderate	6
6	Y. S. Kim 2017	JBI	Yes	Yes	Yes	Yes	Yes	Yes	Yes	Yes	Yes	High	9
7	B. E. Stein 2015	NIH	Yes	Yes	Yes	Yes	Yes	Yes	Yes	No	Yes	High	8
8	J. L. Ellera Gomes 2012	NIH	Yes	Yes	Yes	Yes	Yes	Yes	Yes	No	Yes	High	8
9	S. Y. Lee 2015	NIH	Yes	Yes	Yes	Yes	Yes	Yes	Yes	Yes	Yes	High	9
10	L. N. Muench 2020	NIH	Yes	Yes	Yes	Yes	Yes	Yes	Yes	Yes	Yes	High	9
11	C. H. Jo 2020	NIH	Yes	Yes	Yes	Yes	Yes	Yes	Yes	Yes	Yes	High	9
12	A. Wang 2015	NIH	Yes	Yes	Yes	Yes	Yes	Yes	Yes	Yes	Yes	High	9
13	M. Khoury 2021	NIH	Yes	No	Yes	Yes	No	Yes	Yes	Yes	Yes	High	8
14	K. Tate-Oliver 2013	NIH	Yes	Yes	CD	Yes	Yes	No	Yes	No	No	Low	5
15	C. J. Centeno 2015	NIH	CD	Yes	Yes	No	No	Yes	No	Yes	Yes	Moderate	5
16	A. Singh 2014	NIH	Yes	No	No	Yes	No	No	No	Yes	No	Low	3
	**RCTs (Cochrane Rob 2 tool)**	Randomization process	Deviations from the intended interventions			Missing outcome data		Measurement of the outcome		Selection of the reported result			The overall risk of bias
1	J. L. Hurd 2020	+	–			+		+		+			+
2	G. Rodas 2021	+	–			+		+		+			+
3	D. A. V. Rosário 2021	+	–			–		+		+			؟
4	A. W. Clarke 2011	+	+			+		+		+			+
5	Usuelli, F. G 2017	+	؟			+		+		+			+
6	J. R. Lamas 2019	+	–			–		–		–			–

*Abbreviations*: *CD* Cannot determine, *NR* Not reported, *NA* Not available, *NIH* National Institutes of Health quality assessment tool for case-series studies, *JBI* Joanna Briggs Institute critical appraisal checklist for quasi-experimental studies, *RCT* Randomized clinical trials

### Outcomes

The conclusions of the included studies are presented in Table 1, and the raw results in table S7. All the studies, as previously mentioned, included direct whole-cell injection to the injury site (tendon damage site), and studies that injected extracts from the cells like secretome or biologics (not a “whole-cell”) were excluded. Only Lee et al. [36] used allogenic cells.

### Rotator cuff

All studies, except one [35], showed the safety of cell injection in rotator cuff tendinopathies without serious adverse effects [14, 28–30, 32, 34, 37]. The RCT by Lamas et al. on autologous MSCs in a xenogenic scaffold (OrthADAPT™) for repairing full-thickness rotator cuff tears was terminated because both groups showed adverse effects [35]; 1 patient (8%) in the control group (Scaffold) and 3 patients (23%) in the intervention group (Scaffold + MSC) experienced postoperative complications. Supraclavicular cysts and subacromial inflammatory tissue were observed in these patients. About 60% of both groups experienced re-rupture. The complications experienced in the 2 study groups were not associated with the autologous MSCs, but rather with the scaffold (OrthADAPT™) [35].

Cells used in the rotator cuff repair studies consist of the following type of cells: BMMC [28], bone marrow-derived mesenchymal stem cells (BM-MSCs) [14, 35], adipose-derived mesenchymal stem cells (AD-MSC) [30, 33], bone marrow concentrate (BMC) [25, 32, 37], and uncultured, autologous, fresh, unmodified, adipose-derived regenerative cells (UA-ADRCs) [29].

The included studies have demonstrated that cell injection in tendon disorders yielded beneficial effects. Four studies claimed that cell therapy was a more efficient treatment compared to the control group (MSC and UA-ADRCs) [14, 29, 32, 35], with a lower rate of retear in surgical patients [14, 34], less pain, and higher function [29, 32, 35]. However, Kim YS et al. observed no differences between the cell therapy and control groups in terms of pain, ROM, and functional scores at the final follow-up (28 months) [34]. Nevertheless, the retear rate in MRI was significantly lower (28.5% vs. 14.3% retear; *P* < 0.001) in the cell therapy group and better outcomes were observed in this group at earlier follow-ups. In another study, Kim SJ et al. reported no significant reduction in tear size in the study groups, although substantial improvement was observed in pain and function in the cell injected group [32].

### Elbow

All studies showed the safety of cell injection in elbow tendinopathies without serious or clinically significant adverse effects [27, 31, 36, 41, 44]. However, Lee et al. reported a minor effusion in the elbow joint in 2 of the 12 patients with recalcitrant lateral elbow tendinopathy 52 weeks after allogenic AD-MSC injection [36]. Khoury et al. followed 18 patients with recalcitrant lateral elbow tendinopathy for 6 months after autologous AD-MSC injection; they observed a subcutaneous hematoma at the injection site in 2 participants [31]. In elbow tendon repair studies, the following types of cells are used: AD-MSCs [31, 36], autologous tenocytes [44], and tenocyte-like cells [27].

In addition, all the studies demonstrated the effectiveness of cell injection in elbow tendon disorders [27, 31, 36, 41, 49]. Lee et al. studied the two groups of low-dose (10^6^ cells) and high-dose (10^7^ cells) allogenic AD-MSC injection in patients with lateral elbow tendinopathy, and found no significant differences in function and pain between the groups. However, the improvement in pain and function was faster in the high-dose group, which illustrates the efficacy of cell therapy [36].

### Achilles

All studies demonstrated that cell injection in Achilles tendinopathies was safe without serious adverse effects during the follow-up periods [15, 42, 43]. Moreover, all the studies demonstrated the beneficial effects of cell injection in tendon disorders. Cells used in the Achilles repair studies consist of the following type of cells: BMC [42], adipose-derived tissue stromal vascular fraction (AD-tSVF) [43], and stromal vascular fraction (SVF) [15].

Tate-Oliver and Alexander [43] administered AD-tSVF to 3 patients with Achilles tendinosis and interstitial tears, and Stein et al. [42] augmented 27 Achilles tendon tears with BMC injection. Neither study had a control group [42, 43], and the results of both demonstrated a reduction in pain [42, 43], no retear or re-occurrence [42, 43], structural improvement in the damaged tendon, and the ability to do light activities or return to sports [42, 43] in patients compared to pre-treatment. Findings of the only RCT on recalcitrant non-insertional Achilles tendinopathy treated with SVF (intervention) or platelet-rich plasma (PRP; controls) [15] showed a significant reduction in the VAS pain score and improvement of functional scores (VISA-A and AOFAS) in both groups compared to baseline. No significant differences were detected in the final follow-up (6 months), but the SVF group improved faster. This means that the SVF group participants showed significantly better outcomes in the shorter follow-ups (15 and 30 days follow-up). Radiological data (MRI and US) showed no improvement in either group [15].

### Patellar

All studies showed the safety of cell injection in patellar tendinopathies with no serious adverse effect [26, 38, 39]. Nevertheless, Rodas et al. study comparing BM-MSC and Lp-PRP injections on patients with chronic patellar tendinopathy reported a few mild side effects (one in each group), mostly musculoskeletal such as myalgia and arthralgia [39]. Cells used in patellar tendon repair studies consist of the following type of cells: tenocyte-like cells [26], bone marrow mononuclear cell (BM-MNC) [38], and MSC [39].

All the studies demonstrated the beneficial result of cell injection in patellar tendon disorders [26, 38, 39]. Rodas et al., in their RCT, compared BM-MSC treatment with Lp-PRP treatment as a control group in patients with refractory patellar tendinopathies [39]. They concluded that both treatments successfully reduce pain and improve the VISA-P score with no significant difference. Nevertheless, the BM-MSC group was superior in terms of structural healing in the ultrasound and MRI imaging. In another RCT by Clarke et al. on patients with refractory patellar tendinosis, the cell therapy group (tenocyte-like collagen-producing cells) was superior to the control group (autologous plasma alone) in terms of VISA score with a faster response [26].

### Gluteal

There was only one study on gluteal tendinopathy, in which they used BMC in the intervention group and corticosteroid in the control group [40]. This technique was safe and effective in significantly reducing the VAS score and Lequesne score compared to the control group.

## Discussion

This systematic review investigated the safety and efficacy of cell therapy in treating tendon disorders. Almost all included studies reported the safety of cell injection in their follow-up period with no significant side effects or immunologic reactions. They noted only a few related minor adverse events in some cases (including pain or swelling at the site of the injection [15, 25, 31, 34, 36], abdominal pain [29], musculoskeletal pain [29, 39], upper respiratory tract infection [29], mild effusion of joint [36], and subcutaneous hematoma [15, 31]).

All the studies in this review demonstrated the potential effect of cell therapy in tendon disorder treatment. Although some of the articles reported the beneficial impact of cell injection on tendinopathies and the superiority of the cell injected group compared to controls [14, 26, 28, 29, 32, 40], other RCTs and studies with a control group showed no improvement in outcomes in the treatment group compared to controls [15, 34, 39]. However, the procedure satisfied a high rate of patients [32, 38]. Our results are in line with that of the meta-analysis by Cho et al.; they reviewed only prospective studies on MSC administration in tendinopathies [13]. They analyzed 4 prospective studies and revealed a significant pooled effect size with a significant cell dose-dependent response in pain reduction.

The exact mechanism of action of the MSC effect in tendon healing is still not clear. Studies have suggested that injected stem cells survive for some weeks in the defect [50], differentiate into tenocytes [11, 51], and excrete their secretome (paracrine effect) with regenerative effects [52, 53]. Another possible mechanism is that the MSCs, on their own, release extracellular factors and cytokines, thus accelerating regeneration and modulating immune cell response [53, 54]. Considering that inflammation has a critical role in the tendon tissue damage process, the regulatory effect of MSC can potentially affect tendon tissue repair [53–55].

There are still concerns regarding the safety of cell injection. In previous studies with different settings, such as ischemic cardiomyopathy [56] and myocardial infarction [57], the systemic administration of both allogeneic and autologous MSC appeared safe with minimal adverse effects, including immunologic reactions [13, 56–60]. In line with the present study findings, other systematic reviews on tendon tissue cell therapy did not report serious adverse events in clinical and preclinical studies [13, 18, 61, 62]. However, van den Boom et al., in an article systematically reviewing the efficacy of stem cell therapy in tendon disease treatment, highlighted the potentially harmful consequences of stem cell application such as the development of malignancies in the target organ [9]. Injection of autologous hematopoietic stem cells caused tumor growth in the injected kidney of a patient with renal failure [63], and intrathecal injection of MSCs caused glioma growth in another patient with an ischemic stroke [64]. Major complications of stem cell therapy were observed in other tissues, such as infection following the receipt of umbilical cord blood-derived stem cells [65], tumor formation at the target tissue [63, 64], and worsening of the disease course in patients with macular degeneration [9]. Regarding the complications of cell therapy in tendon tissue, ectopic bone formation was observed in the rabbit model as a result of using MSC [66]. Donor site morbidity when retrieving a sufficient amount of cells is another drawback [67]. Generally speaking, studies on human tendon tissue have not illustrated any major adverse event as a result of the delivering of cells to the tendinopathy site.

Numerous studies have investigated the efficacy of cell therapy in rotator cuff conditions. As far as the available evidence indicates, cell therapy for rotator cuff tendon seems beneficial for the augmentation of rotator cuff repair surgery and for patients with partial-thickness tears who did not respond to conservative medication or physical therapy for more than 3-6 months. Liu et al., in a systematic review study in 2019, evaluated stem cell application in rotator cuff healing [61]. Although only 3 of the articles included in their study were on human subjects, their meta-analysis revealed that VAS and ASES scores at 3 months are more favorable in the stem cell group. Regarding the animal studies included in their review [61], no significant differences were observed between groups when biomechanical evaluation of the tendon was performed. However, motion analysis scores (walking distance, fast walking time, and mean walking speed) were higher in the stem cell group [61]. Our finding revealed that 4 trials favoured cell therapy [14, 29, 34, 35]; however, 1 trial revealed no superiority for the cell group over controls [34].

Lamas et al., in a double-blind RCT (only abstract) [68], assessed the safety and efficacy of autologous MSC administration accompanied by surgical repair in full-thickness rotator cuff tears. The stem cell group (*N* = 8) showed an improvement of 31 points in the Constant score after a year, which was significantly higher than the control group (*N* = 5) with an improvement of 16 points. Other assessments were comparable (VAS pain, retear rate, and repair integrity) [68]. However, 37.5% of the treatment group and 25% of the controls presented with swelling, pain, and retear requiring reoperation. Therefore, the complications of the procedure mandate further RCTs. Lamas et al. published a double-blind RCT in 2019, 4 years after their first study, on the efficacy and safety of autologous MSCs implanted in a xenogenic scaffold in repairing full-thickness rotator cuff tear [35]. They stopped the study due to adverse events in both groups (~ 60% retear) [35], which indicates that the technique of xenogenic scaffold use (OrthADAPT™) should be revised. The complications experienced in the 2 study groups (Scaffold vs. Scaffold + MSC) were not associated with autologous MSCs, but rather with the scaffold (OrthADAPT™) [35]. However, the treatment group showed a significant improvement in the Constant score compared to baseline even though it is inconclusive [35].

No well-designed RCT exists on cell therapy in elbow conditions and epicondylitis. In the elbow tendon disorders, the cell therapy again could be used as a non-surgical rescue treatment after failed first-line options for patients suffering from a partial-thickness tear of extensor tendons. Yet, the existing case studies show an improvement in pain, function, and radiology assessment in patients with refractory lateral epicondylitis [27, 31, 36, 44] and patients with no history of treatment [41]. In the study by Khoury et al. on 18 patients with recalcitrant lateral elbow tendinopathy, pain reduction and improved function were witnessed after injection of autologous ASCs under US guide [31]. Moreover, structural healing was verified using MRI radiology. The use of allogenic AD-MSC in another trial also had comparable results and was safe for patients in terms of immunologic rejection [36]. The advantage of allogenic cell application is that there is no need to harvest cells from the patients individually. The application of cultured autologous tenocytes has the same outcome [44, 49].

Skin-derived tenocyte-like cells were used by Connell et al. to treat refractory lateral epicondylitis [27]. Symptoms relief and structural healing, and no retear were observed in their 12 participants [27]. In the study by Singh et al., BMC, which mainly consists of BM-MSC, was used in patients with lateral epicondylosis, and was found to be effective in terms of the Patient-Rated Tennis Elbow Evaluation (PRTEE) score after 3 months [41]. No comparative clinical trial has been undertaken to determine which of the abovementioned cell types is more efficient in clinical use. Furthermore, no study has compared cell therapy with a control group. Thus, further investigations are necessary to approve cell therapy as a treatment option.

Cell therapy might also improve Achilles tendinopathies. However, Achilles tendinopathy patients are very heterogeneous, and no recommendation can be drawn from them. The RCT by Usuelli et al. revealed the safety of adipose-derived SVF injection for chronic Achilles tendinopathy with minor adverse effects at the site of the adipose tissue harvest [15]. Although both groups of SVF and PRP showed healing effects in the treatment of Achilles tendinopathy, the stem cell group recovered faster [15]. No other studies exist concerning cell injection in the treatment of the human Achilles tendon disorder. Superb results with early weight-bearing and no retear were observed in other case studies on BMC plus surgical tendon repair [42, 43].

Preclinical investigations on rats have presented promising results [69–73]. Okamoto et al. compared the ultimate failure load of the Achilles tendon among the BMC treatment, MSC treatment, and non-treated groups. The BMC group showed greater improvement at the final stage and that possibly the other hematopoietic stem cells are responsible for the better function of MSCs [71]. Machova Urdzikova et al. treated collagenase-induced Achilles with human MSCs and compared them to controls [70]. The MSC group illustrated a more organized ECM structure and vascularization and were safe in the rats [70]. Yao et al. also noted the faster healing of the rat’s Achilles tendon using sutures seeded with bone marrow-derived stem cells [74]. Chong et al. only reported histological and biomechanical improvement of the Achilles tendon in the early stage of administrating BM-MSCs to the transacted tendon in the rabbit model [69].

Cell injection for patellar tendinopathy is not well studied. Likewise shoulder and elbow, chronic patellar tendinopathies that do not respond to nonoperative treatment or rehabilitation for more than 3-6 months should be considered for cell therapy. Two RCTs [26, 39] on the use of BM-MSC and tenocyte-like cell (derived from dermal fibroblast) showed satisfactory healing of the tendon tissue. In the study by Rodas et al. on 20 patients with chronic patellar tendinopathy, although clinical outcomes of the cell group (BM-MSC) and active control group (Lp-PRP) were similar, the structural regeneration in radiology was only observed in the cell group [39]. Thus, the cell group may benefit more in the long term.

Using dermal fibroblast for tendon engineering has been discussed, and the potential positive effect has been established in preclinical studies [75]. These cells were harvested with minimal donor site morbidity and showed tendon regeneration in animal studies [75]. In a human study by Connell et al. [27], tenocyte-like cells derived from skin fibroblasts were injected safely to treat patients with lateral epicondylitis, resulting in symptom subsidence in 11 of the 12 patients and structural healing in the US.

This systematic review faced serious limitations, mainly due to the poor design of the included articles. Many studies included in the review lack control groups, and the ones containing control groups are on a small population, thus leading to the limited power of the studies. More than half of the included studies followed the patient for a year or less, and many for only 3-6 months, which is insufficient to provide compelling evidence. The authors recommend the follow-up of the patients for a more extended time using radiological and laboratory modalities to ensure the procedure’s safety. Studies used various types of cells from diverse sources, bone marrow, adipose tissue, or skin. Many studies in this review administered BMC [14, 15, 25, 32, 33, 37, 38, 40, 42, 43], which contains MSCs, but also contains other biologic factors and platelets. Furthermore, an unknown number of cells were injected. In future research with accurate cell counts, the optimum dose and hazardous dose of MSC injections and other types of cell injections can be determined. Moreover, in most of the included studies, patients who had refractory tendon disease were studied. If milder cases were recruited in future studies, the results could be more representative. In addition, the quality assessment of the studies was performed using three different tools, which may lead to a biased comparison among them. Finally, in many cases, surgical groups were compared with non-surgical groups, and these two types of patients represent two different populations.

## Conclusion

According to the clinical studies in this systematic review, cell-based therapy for different tendinopathies appears to be safe. Numerous studies have demonstrated the potential benefits of cell injection in tendinopathy treatments, but there are currently no convincing RCTs with large sample sizes and sufficient follow-up intervals to demonstrate their effectiveness conclusively. As far as the available evidence indicates, cell therapy for rotator cuff tendon seems beneficial for the augmentation of rotator cuff repair surgery and for patients with partial-thickness tears who did not respond to conservative medication or physical therapy for more than 3-6 months. In the elbow tendon disorders, the cell therapy again could be used as a non-surgical rescue treatment after failed first-line options for patients suffering from a partial-thickness tear of extensor tendons. Likewise, chronic patellar tendinopathies that do not respond to nonoperative treatment or rehabilitation for more than 3-6 months should be considered for cell therapy. However, Achilles tendinopathy patients are very heterogeneous, and no recommendation can be drawn from them. Based on available evidence, cell therapy should be suggested in specific conditions at each site.

## Supplementary Information

**Additional file 1.** PRISMA checklist.

**Additional file 2: Table S1.** PubMed keywords and Mesh terms. **Table S2. **Databases search results. **Table S3. **PubMed databases search details. **Table S4. **PICOT table. **Table S5. **Inclusion and Exclusion criteria. **Table S6. **Excluded case reports. **Table S7. **Studies’ outcomes and results.

## Data Availability

Not applicable.
