# Supplementary material for: Cell therapy efficacy and safety in treating tendon disorders: a systemic review of clinical studies
Source: J Exp Orthop. 2022 Aug 30;9:85. doi: 10.1186/s40634-022-00520-9 (PMC9428081; doi:10.1186/s40634-022-00520-9)
Supplement: Supplementary file 2 — Additional file 2: Table S1. PubMed keywords and Mesh terms. Table S2. Databases search results. Table S3. PubMed databases search details. Table S4. PICOT table. Table S5. Inclusion and Exclusion criteria. Table S6. Excluded case reports. Table S7. Studies’ outcomes and results. [file 40634_2022_520_MOESM2_ESM.docx]

***Supplementary file***

**Title: Cell Therapy Efficacy and Safety in Treating Tendon Disorders: A Systemic Review of Clinical Studies**

**Authors List:** Seyed Peyman Mirghaderi, Zahra Valizadeh, Kimia Shadman, Thibault Lafosse M.D., Leila Oryadi-Zanjani M.D., Mir Saeed Yekaninejad Ph.D., Mohammad Hossein Nabian M.D.

**Affiliation:**

Center of Orthopedic Trans-Disciplinary Applied Research (COTAR), Tehran University of Medical Sciences, Tehran, Iran

Correspondence: [dr.nabian@gmail.com](mailto:dr.nabian@gmail.com)

Journal of Experimental Orthopaedics

**Supplemental tables**

S1: PubMed keywords and Mesh terms

S2: Databases search results

S3: PubMed databases search details

S4: PICOT table

S5: Inclusion and Exclusion criteria

S6: Excluded case reports

S7: Studies' outcomes and results

**Table S1** PubMed keywords and Mesh terms

| **Component 1 (Cell therapy)** | **AND** | **Component 2 (Tendon)** |
| --- | --- | --- |
| "Stem Cells"[Mesh] OR |  | “Tendons”[Mesh] OR |
| “Stem Cell*”[Title/Abstract] OR |  | “Tendon*”[Title/Abstract] OR |
| “Progenitor Cell*”[Title/Abstract] OR |  | “Tenogen*”[Title/Abstract] OR |
| “Mother Cell*”[Title/Abstract] OR |  | “Epitenon*”[Title/Abstract] OR |
| “Colon-Forming Unit*”[Title/Abstract] OR |  | “Paratenon*”[Title/Abstract] OR |
| “Colony Forming Unit*”[Title/Abstract] OR |  | “Patella*”[Title/Abstract] OR |
| “Fibroblast*”[Title/Abstract] OR |  | "Tendinopathy"[Mesh] OR |
| “Tenoblast*”[Title/Abstract] OR |  | "Tendin*"[Title/Abstract] OR |
| “Tenocyte*”[Title/Abstract] OR |  | "Enthes*"[Title/Abstract] OR |
| "Cell Therapy"[Title/Abstract] OR |  | “Endotenon*”[Title/Abstract] OR |
| “Tenogenic Cell*”[Title/Abstract] OR |  | "Achilles Tendon"[Mesh] OR |
| "Cell- And Tissue-Based Therapy"[Mesh] OR |  | "Hamstring Tendons"[Mesh] OR |
| "Cell Therap*"[Title/Abstract] OR |  | "Patellar Ligament"[Mesh] OR |
| "Cell-Therap*"[Title/Abstract] OR |  | "Rotator Cuff"[Mesh] OR |
| "Multipotent Stem Cells"[Mesh] OR |  | Patella*[Title/Abstract] OR |
| "Mesenchymal Stem Cells"[Mesh] OR |  | Rotator Cuff*[Title/Abstract] OR |
| "Stromal Cell*" Title/Abstract] OR |  | Teres Minor*[Title/Abstract] OR |
| "Mesenchymal Stem Cell*"[Title/Abstract] OR |  | Subscapularis*[Title/Abstract] OR |
| "Mesenchymal Stromal Cell*"[Title/Abstract] OR |  | Infraspinatus*[Title/Abstract] OR |
| "Wharton Jelly"[Title/Abstract] OR |  | Supraspinatus*[Title/Abstract] OR |
| "Wharton’s Jelly"[Title/Abstract] OR |  | "Tenosynovitis"[Mesh] OR |
| "Whartons Jelly"[Title/Abstract] OR |  | Tenosynov*[Title/Abstract] OR |
| "Stromal Vascular Fraction*"[Title/Abstract] OR |  | "Elbow Tendinopathy"[Mesh] OR |
| "Stromal Vascular Fraction"[Mesh] OR |  | Epicondylit*[Title/Abstract] OR |
| Svf*[Title/Abstract] |  | Golfer's Elbow*[Title/Abstract] OR |
|  |  | Golfers Elbow*[Title/Abstract] OR |
|  |  | "Tennis Elbow"[Mesh] OR |
|  |  | Tennis Elbow*[Title/Abstract] OR |
|  |  | "Enthesopathy"[Mesh] OR |
|  |  | Enthesopath*[Title/Abstract] OR |
|  |  | "Tendon Injuries"[Mesh] OR |
|  |  | Tendon Injur*[Title/Abstract] OR |
|  |  | "Rotator Cuff Injuries"[Mesh] |

**Table S2** Databases search results

| **Database Name** | **Platform** | **Date Coverage** | **Dates of Search** | **# of results** | **Alert** |
| --- | --- | --- | --- | --- | --- |
| 1. Scopus | Elsevier | 1788-present | 22/03/2021  26/12/2021 | 1806 | yes |
| 2. Pubmed | NLM | Inception-2021 | 22/03/2021  26/12/2021 | 1081 | yes |
| 3. Web of science |  | 1990-2021 | 22/03/2021  26/12/2021 | 1437 | no |
| 4. Embase | Elsevier | Inception-2021 | 22/03/2021  26/12/2021 | 1667 | yes |
| 5. Cochrane | Wiley | Inception-2021 | 22/03/2021  26/12/2021 | 5 | yes |
| Hand search | - | - | - | 21 | - |

**Total Records = 6017**

**Total Records after deduplication = 2543**

**Table S3** PubMed databases search details

| **Pubmed (NML) search details** |
| --- |
| Date of Search: March 22, 2021  Number of results: 1062  Date of Search: Dec 26, 2021  Number of results: 1081 (+19) |
| **Search query:**  ("Stem Cells"[Mesh] OR "Stem Cell*"[Title/Abstract] OR "Progenitor Cell*"[Title/Abstract] OR "Mother Cell*"[Title/Abstract] OR "Colon-Forming Unit*"[Title/Abstract] OR "Colony Forming Unit*"[Title/Abstract] OR "Fibroblast*"[Title/Abstract] OR "Tenoblast*"[Title/Abstract] OR "Tenocyte*"[Title/Abstract] OR "Cell Therapy"[Title/Abstract] OR "Tenogenic Cell*"[Title/Abstract] OR "Cell- And Tissue-Based Therapy"[Mesh] OR "Cell Therap*"[Title/Abstract] OR "Cell-Therap*"[Title/Abstract] OR "Multipotent Stem Cells"[Mesh] OR "Mesenchymal Stem Cells"[Mesh] OR "Stromal Cell*" Title/Abstract] OR "Mesenchymal Stem Cell*"[Title/Abstract] OR "Mesenchymal Stromal Cell*"[Title/Abstract] OR "Wharton Jelly"[Title/Abstract] OR "Wharton’s Jelly"[Title/Abstract] OR "Whartons Jelly"[Title/Abstract] OR "Stromal Vascular Fraction*"[Title/Abstract] OR "Stromal Vascular Fraction"[Mesh] OR Svf*[Title/Abstract]) AND ("Tendons"[Mesh] OR "Tendon*"[Title/Abstract] OR "Tenogen*"[Title/Abstract] OR "Epitenon*"[Title/Abstract] OR "Paratenon*"[Title/Abstract] OR "Patella*"[Title/Abstract] OR "Tendinopathy"[Mesh] OR "Tendin*"[Title/Abstract] OR "Enthes*"[Title/Abstract] OR "Endotenon*"[Title/Abstract] OR "Achilles Tendon"[Mesh] OR "Hamstring Tendons"[Mesh] OR "Patellar Ligament"[Mesh] OR "Rotator Cuff"[Mesh] OR Patella*[Title/Abstract] OR Rotator Cuff*[Title/Abstract] OR Teres Minor*[Title/Abstract] OR Subscapularis*[Title/Abstract] OR Infraspinatus*[Title/Abstract] OR Supraspinatus*[Title/Abstract] OR "Tenosynovitis"[Mesh] OR Tenosynov*[Title/Abstract] OR "Elbow Tendinopathy"[Mesh] OR Epicondylit*[Title/Abstract] OR Golfer's Elbow*[Title/Abstract] OR Golfers Elbow*[Title/Abstract] OR "Tennis Elbow"[Mesh] OR Tennis Elbow*[Title/Abstract] OR "Enthesopathy"[Mesh] OR Enthesopath*[Title/Abstract] OR "Tendon Injuries"[Mesh] OR Tendon Injur*[Title/Abstract] OR "Rotator Cuff Injuries"[Mesh]) |

**Table S4** PICOT

| **PICOT** | |
| --- | --- |
| **Participants** | Adults with any Tendon pathologies |
| **Intervention** | Any cell therapy administration |
| **Comparison** | Other treatment, no treatment, placebo, or no control group |
| **Outcome** | Clinical, Functional, and radiological outcomes |
| **Type** | Randomized clinical trials (RCT), non-RCT, and case series |

**Table S5** Inclusion and Exclusion criteria

| Inclusion criteria | Exclusion criteria |
| --- | --- |
| 1- Adults more than 18 with tendon disorders that leads to pain or reduced function  2- Intervention group treated with cell therapy  3- any clinical study  4- Studies that include primary outcome measures for pain intensity, for example, visual analogue scale (VAS), disability, function (like ASES), range of motion (ROM), quality of life, or radiological assessment | 1- non-human studies  2- non-English studies  3- review articles  4- case reports, congress abstracts, commentaries studies, and book chapters  5- Studies not published in any peer-reviewed journals (such as Clinicaltrials.gov)  6- Studies about non-musculoskeletal disorders (neurological disorders visceral pain, referral pain, neoplasm, visceral disorders) |

***Table S6*** Excluded relevant case reports

| **ID** | **Study (first author, year, reference)** | **Country** | **Study design** | **Type and Site of injury** | **Groups and population** | **Gender (male)** | **Mean age ± SD y.** | **Cell type** | **Cell source** | **Number cell administered** | **Follow up (SD) months** | **Surgical techniques** | **Conclusion** | **Level of evidence** |
| --- | --- | --- | --- | --- | --- | --- | --- | --- | --- | --- | --- | --- | --- | --- |
| 2 | J. Freitag 2020 (37) | Australia | Case report | Elbow CEO tendinosis (right hand) | AD-MSC + PRP (N=1) | N=1 | 52 | Autologous AD-MSC | Adipose tissue (abdominal subcutaneous fat) | 1.0x10^7^ | 30 | No surgery | Pain and functional improvement after AD-MSC therapy, No significant adverse events | 4 |
| 3 | K. A. Farina 2021 (38) | USA | Case report | Partial Achilles tendon tear | BMC (N=1) | N=0 | 25 | Autologous MSC | BMC (from posterior iliac crest) | - | 8 | No surgery | Tear of Achilles tendon showed satisfactory healing in response to BMC injection after 8 months. | 4 |
| 4 | S. Giannotti 2017 (39) | Italy | Case report | Extensor mechanism allograft failure after TKA | BMC+PRF (N=1) | N=0 | 86 | Autologous MSC | BMC (from anterior iliac crest bone) | - | 6 | Extensor mechanism allograft reconstruction | Concentrated marrow cells with PRF speed the healing process of the allograft. | 4 |

| ***Table S7***  Studies' outcomes and results, P demonstrated the significance between the time point and before the intervention, P* shown the significance between the case and control groups | | | | | | | | | | |
| --- | --- | --- | --- | --- | --- | --- | --- | --- | --- | --- |
| **Study (first author, year, reference)** | **Outcome measure** | **Groups and population** | **Outcome** | | | | | | | **Conclusion** |
|  |  |  | Before intervention | 4-6 weeks | 2-3 months | 6 months | 12 months | 24 months ≤ | Other results |  |
| **Rotator Cuff** | | | | | | | | | | |
| Ellera Gomes 2011 | MRI | BMMC group (N=14), no controls |  |  |  |  | Low signal intensity areas along the supraspinatus tendon and distal muscle belly (N=8/14), high-intensity blooming small round artifact at the bursal and tendon topography (N=11/14), Formation of a high-signal intensity zone at the critical zone (N=6/14) None of these findings affected the final functional Result | | | BMMC implantation in a patient with rotator cuff sutures is safe and has promising results compared to historical data (patients underwent surgical procedures without stem cells application). |
|  | UCLA |  | 12 ± 3 |  |  |  | 31 ± 3.2 |  |  |  |
| Hernigou P 2014 | Failure (Re-tear/nonhealing) | MSC group (n=45) |  | 0% |  |  |  | 56% (N=25) (p<0.05) |  | More prominent and earlier (2 months earlier, p=0.04) healing in MSC-treatment group, total healing more likely achieved when MSCs> 2500 cell.mL-1 |
|  |  | Control group (n=45) |  | 33% |  |  |  | 13% (N = 6) (receiving low number of MSCs), 87% (N=39) intact rotator cuff | 4 times more likely to show a poor outcome |  |
|  | healing surface (cm^2^) |  |  |  | 22.5±18.9 mm^2^ |  |  |  | MSCs number was correlated to the surface healing (correlation coefficient = 0.6) | Injection of BMC–PRP to the rupture site improve the clinical symptoms and reduction of tear size |
| C. J. Centeno 2015 | DASH | BMC+PRP+PL (N=81 arm) | 36.1 ± 19.8 | 18.5 |  | 17.1 ± 12.6 (P<0.001) |  | 3.3 | no serious adverse event, adverse event =5 (4.9%) | Rotator cuff tear patients treated with BMC showed significant pain and functional improvement |
|  | NPS |  | 4.3±2.2 | 2.6 |  | 2.4 ± 2.1 (P<0.001) |  | 1.5 |  |  |
|  | self-rated improvement |  |  |  |  |  | 0.48 |  |  |  |
| Kim, Y. S 2017 | VAS at rest | AD-MSC +arthroscopic repair (N=35) | 1.9± 0.8 |  |  |  |  | 0.4 ±0.6 , P<0.001, |  | AD-MSC injection along with rotator cuff repair reduce retear rate significantly, but no clinical differences at the end of the follow up comparing to controls |
|  |  | arthroscopic repair (N=35) | 2.1± 0.9, P*=0.456 |  |  |  |  | 0.3± 0.5 , P<0.001, P*=0.256 |  |  |
|  | VAS during motion | AD-MSC +arthroscopic repair | 5.3± 1.2 |  |  |  |  | 2.4 ±1.1 P<0.001 |  |  |
|  |  | arthroscopic repair | 6.5± 1.5, P*=0.572 |  |  |  |  | 2.1± 0.9 P<0.001 , P*=0.776 |  |  |
|  | Constant score | AD-MSC +arthroscopic repair | 65.2± 14.6 |  |  |  |  | 78.3 ±14.9 P<0.001 |  |  |
|  |  | arthroscopic repair | 63.3± 15.3, P*=0.871 |  |  |  |  | 80.1± 13.2 P<0.001 , P*=0.634 |  |  |
|  | UCLA | AD-MSC +arthroscopic repair | 26.5± 5.2 |  |  |  |  | 29.8 ±5.1 , P=0.037 |  |  |
|  |  | arthroscopic repair | 25.1± 5.6 , P*=0.841 |  |  |  |  | 30.5± 4.8 , P=0.013 , P*=0.302 |  |  |
|  | ROM Forward flexion, deg | AD-MSC +arthroscopic repair | 148.6± 19.9 |  |  |  |  | 155.2 ±25.3 , P=0.041 |  |  |
|  |  | arthroscopic repair | 145.2± 21.5 , P*=0.837 |  |  |  |  | 156.4± 23.2 , P=0.002 , P*=0.921 |  |  |
|  | ROM External rotation, deg | AD-MSC +arthroscopic repair | 52.3± 19.4 |  |  |  |  | 65.5±23.4 , P=0.008 |  |  |
|  |  | arthroscopic repair | 53.9± 18.9 , P*=0.809 |  |  |  |  | 67.2± 21.5 , P=0.016 , P*=0.761 |  |  |
|  | ROM Internal rotation, deg | AD-MSC +arthroscopic repair | T 11.3 |  |  |  |  | T 11.1 , P=0.834 |  |  |
|  |  | arthroscopic repair | T 10.5, P*=0.235 |  |  |  |  | T 9.8 , P=0.625, P*=0.192 |  |  |
|  | Complete healing (MRI) | AD-MSC +arthroscopic repair |  |  |  |  |  | 30 (85.7) |  |  |
|  |  | arthroscopic repair | P^*^<0.001 |  |  |  |  | 25 (71.4) , P*<0.001 |  |  |
|  | Retear (MRI) | AD-MSC +arthroscopic repair |  |  |  |  |  | 5 (14.3) |  |  |
|  |  | arthroscopic repair | P^*^<0.001 |  |  |  |  | 10 (28.5), P*<0.001 |  |  |
|  | Partial-thickness (MRI) | AD-MSC +arthroscopic repair |  |  |  |  |  | 2 (5.7) |  |  |
|  |  | arthroscopic repair | P^*^<0.001 |  |  |  |  | 1 (2.8), P*<0.001 |  |  |
|  | Full-thickness (MRI) | AD-MSC +arthroscopic repair |  |  |  |  |  | 3 (8.6) |  |  |
|  |  | arthroscopic repair group | P^*^<0.001 |  |  |  |  | 9 (25.7), P*<0.001 |  |  |
| S. J. Kim 2017 | Tear size by ultrasound | BMC+PRP group (N=12, no control) | 30.2±24.5 mm^2^ |  | 22.5±18.9 mm2 , P<0.05 |  |  |  |  | Injection of BMC–PRP to the rupture site leads to improvement of the reduction of the clinical symptoms of tear size. |
|  | VAS |  | 5.8± 1.9 | 5.0±2.3 | 2.8±2.3 (p<0.01) |  |  |  |  |  |
|  | MMT |  | grade3=3 patients, grade4=4,grade5=5 | grade3=4, grade 4=3, grade 5=5 | grade3=0, grade 4= 5, grade 5= 7 |  |  |  |  |  |
|  | ASES |  | 39.4±13.0 | 52.9±22.9 | 71.8±19.7 (p<0.01) |  |  |  |  |  |
| S. J. Kim 2018 | Tear size by ultrasound | BMC+PRP group (n=12) | 9.7 ± 3.4 | 7.6± 3.2 | 5.9± 2.44 |  |  |  | no side effects or complications | BMC-PRP injection improve VAS and ASES scores (improve pain and shoulder function), tear size changes, and MMT did not statistically differ among groups |
|  |  | Control group (n=12) | 8.9 ± 5.0 mm (P = 0.178) | 7.8± 4.0 (P = 0.235) | 8.1± 3.8 (P = 0.312) |  |  |  |  |  |
|  | VAS | BMC+PRP group | 5.8± 1.9 | 2.3 ± 0.8 | 1.9 ± 0.7 |  |  |  |  |  |
|  |  | Control group | 5.7 ± 1.6 (P = 0.906) | 3.6 ± 2.3 (P = 0.147) | 3.7 ± 1.8 (P = 0.039) |  |  |  |  |  |
|  | MMT | BMC+PRP group | grade3=3 patients, grade4=4, grade5=5 | grade3=0, grade 4=6, grade 5=6 | grade3=0, grade 4= 6, grade 5=6 |  |  |  |  |  |
|  |  | Control group | grade3=2 patients, grade4=1,grade5=9 (P = 0.208) | grade3=0, grade 4= 3, grade 5=9 (P = 0.206) | grade3=0, grade 4= 3, grade 5=9 (P = 0.206) |  |  |  |  |  |
|  | ASES | BMC+PRP group | 39.4±13.0 | 54.5±11.5 | 74.1±8.5 |  |  |  |  |  |
|  |  | Control group | 45.9 ± 12.4 (P = 0.228) | 56.3 ± 12.3 (P= 0.712) | 62.2 ± 12.2 (P = 0.011) |  |  |  |  |  |
|  | Change of the painkiller in frequency and dose | BMC+PRP group |  |  | decrease=6, no change=5, increase=1 |  |  |  |  |  |
|  |  | Control group |  |  | decrease=2, no change=7, increase=3 (P = 0.189) |  |  |  |  |  |
| Hurd, J. L 2020 | AE | UA-ADRCs (N =11) |  |  |  | 3.1 ± 0.5 |  |  |  | UA-ADRCs application in sPTRCT patients is safe and significantly improved function of the shoulder comparing to the control group, with no side effects. |
|  |  | Methylprednisolone(n=5) |  |  |  | 2.6 ±0.8 , P*=0.622 |  |  |  |  |
|  | ASES | UA-ADRCs | 58.7 ± 5.8 |  | 86.1 ± 4.9 | 89.4 ± 4.9 , P<0.05 |  |  |  |  |
|  |  | Methylprednisolone | 50.6 ± 6.7 |  | 60.8 ± 6.2 , P*<0.05 | 68.4 ± 4.4 , P<0.05 , P*<0.05 |  |  |  |  |
|  | RAND Short Form-36 | UA-ADRCs | 557 ± 40.1 |  | 696 ± 15.7 | 691 ± 22.6 , P>0.05 |  |  |  |  |
|  |  | Methylprednisolone | 523 ± 40.4 |  | 586 ± 44.0 , P*>0.05 | 599 ±72.2 , P>0.05 , P*>0.05 |  |  |  |  |
|  | VAS | UA-ADRCs | 2.6 ± 0.7 |  | 0.5 ± 0.2 | 0.9 ± 0.4 , P>0.05 |  |  |  |  |
|  |  | Methylprednisolone | 4.1 ± 1.1 |  | 3.9 ± 1.1 , P*>0.05 | 2.5 ± 0.8 , P>0.05 , P*>0.05 |  |  |  |  |
|  | tear size (mm3, by MRI) | UA-ADRCs | 58.6 ± 11.3 |  | 45.0 ± 6.8 | 44.5 ± 10.3 , P>0.05 |  |  |  |  |
|  |  | Methylprednisolone | 28.7 ± 4.8 |  | 34.6 ± 10.4 , P*>0.05 | 35.0 ± 12.4 , P>0.05 , P*>0.05 |  |  |  |  |
| C. H. Jo 2020 | AE | Low dose (N=3) |  |  |  |  |  |  | No serious adverse event within the 2 years follow-up | Intratendinous injection of AD-MSCs is a safe and effective treatment for partial tears of rotor cuff |
|  |  | Mid dose (N=3) |  |  |  |  |  |  |  |  |
|  |  | High dose (N=13) |  |  |  |  |  |  |  |  |
|  | VAS Pain at rest, mean (95% CI) | Low dose | 3.3 (-0.5-7.1) | 1.7 (-1.2-4.5) , P=0.30 | 2.0 (-3.0-7.0) , P=0.578 | 0.7 (-0.8-2.1) , P=0.157 | 0 , P=0.063 | 0 , P=0.063 |  |  |
|  |  | Mid dose | 4.0 (1.5-6.5) | 3.0 (-3-9.6) , P=0.58 | 2.0 (-3.0-7.0) , P=0.321 | 0.7 (-2.2-3.5) , P=0.109 | 0.1 (-0.4-0.5) , P<0.001 | 0.2 (-0.4-0.8) , P<0.001 |  |  |
|  |  | High dose | 6.1 (5.1-7.1) | 4.3 (2.9-5.6) , P=0.012 | 3.6 (2.6-4.6) , P<0.001 | 1.1 (0.7-1.6) , P<0.001 | 0.9 (0.3-1.5) , P<0.001 | 0.4 (0.1-0.7) , P<0.001 |  |  |
|  | VAS Pain on motion, mean (95% CI) | Low dose | 7.7 (3.9-11.5) | 5.0 (2.5-7.5) , P=0.094 | 4.3 (1.5-7.2) , P=0.109 | 3.7 (2.2-5.1) , P=0.057 | 1.2 (-1.7-4.1) , P=0.022 | 0.8 (-0.2-1.7) , P=0.018 |  |  |
|  |  | Mid dose | 9.0 (6.5-11.5) | 7.3 (5.9-8.8) , P=0.130 | 5.7 (2.8-8.5) , P=0.063 | 3.0 (-1.3-7.3) , P=.059 | 0.7 (-0.8-2.2) , P<0.001 | 0.5 (-0.3-1.4) , P<0.001 |  |  |
|  |  | High dose | 9.0 (8.7-9.4) | 6.6 (5.8-7.4) , P<0.001 | 5.2 (4.2-6.2) , P<0.001 | 2.6 (2.1-3.1) , P<0.001 | 1.6 (0.9-2.2) , P<0.001 | 0.7 (0.3-1.2) , P<0.001 |  |  |
|  | VAS Pain at night, mean (95% CI) | Low dose | 4.0 (-2.6-10.6) | 3.0 (3.0-3.0) , P=0.580 | 3.7 (0.8-6.5) , P=0.885 | 2.0 (-0.5-4.5) , P=0225 | 1.7 (-3.5-6.8) , P=0.073 | 0.3 (-1.1-1.8) , P=0.093 |  |  |
|  |  | Mid dose | 6.7 (5.2-8.1) | 4.3 (-3.3-11.9) , P=0.336 | 2.7 (-3.6-8.9) , P=0.12 | 1.0 (-1.5-3.5) , P=0.014 | 1.4 (-0.6-3.4) , P<0.001 | 0.9 (-0.6-2.4) , P<0.001 |  |  |
|  |  | High dose | 7.7 (6.9-8.5) | 6.2 (5.2-7.1) , P=0.019 | 4.9 (5.2-7.1) , P=0.001 | 2.0 (3.7-6.1) , P<0.001 | 1.5 (0.7-2.2) , P<0.001 | 0.8 (0.2-1.3) , P<0.001 |  |  |
|  | VAS Mean pain, mean (95% CI) | Low dose | 4.0 (0.6-7.4) | 2.8 (2.2-3.4) , P=0.311 | 2.6 (-0.9-6.2) , P<0.465 | 1.4 (0.1-2.7) , P=0.087 | 1.0 (-1.7-3.6) , P=0.024 | 0.4 (-0.3-1.1) , P=0.032 |  |  |
|  |  | Mid dose | 5.8 (4.0-7.7) | 4.2 (-1.9-10.4) , P=0.395 | 2.3 (-2.7-7.4) , P<0.116 | 1.0 (-1.5-3.5) , P=0.014 | 0.8 (-0.5-2.1) , P<0.001 | 0.5 (-0.3-1.3) , P<0.001 |  |  |
|  |  | High dose | 7.2 (6.5-7.9) | 5.5 (4.7-6.4) , P=0.03 | 4.2 (3.2-5.3) , P<0.001 | 1.7(1.3-2.1) , P<0.001 | 1.3 (0.7-1.8) , P<0.001 | 0.7 (0.3-1.0) , P<0.001 |  |  |
|  | VAS Worst pain, mean (95% CI) | Low dose | 7.7 (4.8-10.5) | 5.0 (2.5-7.5) , P=0.157 | 3.7 (0.8-6.5) , P<0.074 | 3.3 (0.5-6.2) , P=0.083 | 2.3 (-1.5-6.1) , P=0.067 | 3.0 (-0.5-4.5) , P<0.001 |  |  |
|  |  | Mid dose | 8.7 (5.8-11.5) | 7.7 (6.2-9.1) , P=0.225 | 6.0 (3.5-8.5) , P=0.157 | 4.0   (-1.3-7.3) , P=0.077 | 2.5 (-0.2-5.2) , P=0.005 | 1.9 (-0.7-4.4) , P<0.001 |  |  |
|  |  | High dose | 9.0 (8.7-9.3) | 7.2 (6.6-7.9) , P<0.001 | 5.4 (4.4-6.3) , P<0.001 | 2.8 (2.3-3.4) , P<0.001 | 2.6 (1.7-3.5) , P<0.001 | 1.5 (0.9-2.1) , P<0.001 |  |  |
|  | range of motion | Low dose | no significant improvement was observed in the other ROM |  |  |  |  |  |  |  |
|  |  | Mid dose |  |  |  |  |  |  |  |  |
|  |  | High dose |  |  |  |  |  |  |  |  |
|  | Forward flexion, deg, mean (95%CI) | Low dose | 148.3 (104.7-192) | 85.0 (-8.8-178.8) , P=0.094 | 145.0 (100.2-189.8) , P<0.184 | 148.3 (117.1-179.6) , P=1.00 | 163.3 (156.2-170.5) , P=0.324 | 160.0 (135.2-184.8) , P=0.118 |  |  |
|  |  | Mid dose | 160.0 (115.2-204.8) | 111.7 (29.0-194.4) , P=0.041 | 155.0 (117.7-192.3) , P<0.225 | 161.7 (142.7-180.6) , P=0.84 | 167.2 (162.9-171.5) , P=0.503 | 166.0 (150.5-181.5) , P=0.654 |  |  |
|  |  | High dose | 160.0 (154.0-166.0) | 97.6 (83.7-111.4) , P<0.001 | 131.6 (117.8-145.4) , P<0.001 | 155.0 (144.2-165.8) , P=0.389 | 163.0 (156.7-169.4) , P=0.352 | 166.2 (159.8-172.5) , P=0.109 |  |  |
|  |  | Low dose | 138.3 (2.1-274.6) | 71.7 (3.3-140.1) , P=0.195 | 141.7 (41.3-242.1) , P<0.728 | 156.7 (120.8-192.5) , P=0.514 | 165.0 (143.5-186.5) , P=0.543 | 166.7 (152.3-181.0) , P=0.423 |  |  |
|  | Abduction, deg, mean (95%CI) | Mid dose | 170.0 (145.2-194.8) | 98.3 (-2.8-199.5) , P=0.06 | 150.0 (63.1-236.9) , P<0.427 | 178.3 (171.2-185.5) , P=0.199 | 174.5 (169.8-179.2) , P0.478 | 168.5 (152.9-184.1) , P=0.902 |  |  |
|  |  | High dose | 166.2 (157.8-174.5) | 80.2 (61.4-99.0) , P<0.001 | 123.5 (100.5-146.5) , P<0.001 | 145.2 (126.6-163.9) , P=0.05 | 164.7 (154.9-174.4) , P=0.781 | 166.0 (159.3-172.7) , P=0.974 |  |  |
|  | External rotation with arm at the side, deg, mean (95%CI) | Low dose | 45.0 (-24.2-114.2) | 21.7 (-25.4-68.7) , P=0.118 | 33.3 (-16.9-83.5) , P=0.118 | 31.7 (12.7-50.6) , P=0.371 | 41.7 (34.5-48.8) , P=0.84 | 40.0 (-3.0-83.0) , P=0.580 |  |  |
|  |  | Mid dose | 41.7 (34.5-48.8) | 25.0 (12.6-37.4) , P<0.063 | 41.7 (-2.0-85.3) , P=1.00 | 38.3 (19.4-57.3) , P=0.635 | 42.7 (34.9-50.4) , P=0.792 | 64.0 (45.6-82.4) , P=0.034 |  |  |
|  |  | High dose | 45.8 (41.2-50.4) | 24.5 (18.9-30.1) , P<0.001 | 27.8 (21.5-34.1) , P<0.001 | 35.4 (24.2-46.5) , P=0.096 | 43.1 (37.1-49.1) , P=0.380 | 58.5 (55.2-61.8) , P<0.001 |  |  |
|  | Internal rotation, vertebral level, mean (95%CI) | Low dose | 9.3 (-2.1-20.8) | 2.3 (-7.7-12.4) , P=0.109 | 10.0 (7.5-12.5) , P=0.789 | 10.0 (7.5-12.5) , P=0.853 | 10.3 (7.5-13.2) , P=0.775 | 12.0 (9.5-14.5) , P=0.49 |  |  |
|  |  | Mid dose | 10.7 (5.5-15.8) | 5.3 (-1.8-12.5) , P=0.047 | 11.3 (8.5-14.2) , P=0.635 | 11.0 (6.0-16.0) , P=0.423 | 11.1 (9.1-13.0) , P0.776 | 11.9 (11.0-12.8) , P=0.378 |  |  |
|  |  | High dose | 10.4 (8.5-12.3) | 5.0 (2.3-5.8) , P=0.003 | 7.2 (5.0-9.4) , P<0.001 | 8.5 (6.7-10.4) , P<0.001 | 10.9 (9.8-12.1) , P<0.001 | 12.1 (11.6-12.5) , P=0.040 |  |  |
|  | Supraspinatus,Ib, mean (95%CI) | Low dose | 9.1 (-3.7-21.9) | 0 , P<0.092 | 5.8 (2.7-9.0) , P=0.287 | 5.7 (1.5-10) , P=0.423 | 8.5 (-0.9-17.8) , P=0.510 | 9.9 (-2.0-21.8) , P=0.321 |  |  |
|  |  | Mid dose | 9.9 (-8.3-28.1) | 5.3 (-8.6-19.1) , P<0.132 | 11.8 (-9.1-32.7) , P=0.478 | 12.5 (-6.0-30.9) , P=0.124 | 10.4 (7.0-13.8) , P=0.914 | 13.9 (9.1-18.6) , P=0.425 |  |  |
|  |  | High dose | 6.5 (4.0-9.0) | 1.1 (-0.0-2.2) , P<0.001 | 4.6 (3.5-5.8) , P=0.095 | 6.5 (5.6-7.3) , P=0.993 | 9.2 (7.0-11.4) , P=0.002 | 11.2 (8.1-14.2) , P<0.001 |  |  |
|  | Infraspinatus, lb, mean (95%CI) | Low dose | 7.9 (-2.3-18.2) | 1.2 (-1.9-4.5) , P=0.064 | 5.1 (-2.5-12.8) , P=0.043 | 5.4 (0.4-10.3) , P=0.231 | 8.0 (-1.2-17.3) , P=0.918 | 9.5 (0.1-18.8) , P=0.022 |  |  |
|  |  | Mid dose | 7.3 (0.5-14.2) | 4.4 (-1.9-10.6) , P=0.087 | 10.3 (-1.9-22.6) , P0.282 | 9.9 (1.8-18) , P=0.056 | 9.4 (6.3-12.4) , P=0.225 | 12.8 (7.4-18.2) , P=0.087 |  |  |
|  |  | High dose | 5.8 (4.0-7.7) | 3.6 (2.3-4.9) , P=0.004 | 5.6 (3.8-7.5) , P=0.815 | 7.1 (5.6-8.6) , P=0.152 | 8.5 (6.6-10.4) , P<0.001 | 9.3 (7.1-11.6) , P<0.001 |  |  |
|  | Subscapularis, lb, mean (95%CI) | Low dose | 13.6 (-3.6-30.8) | 4.2 (-8.1-16.4) , P=0.055 | 11.5 (-11.6-34.6) , P0.51 | 10.0 (-2.4-22.4) , P=0.443 | 10.7 (-4.8-26.3) , P=0.473 | 14.1 (-8.9-37.1) , P=0.809 |  |  |
|  |  | Mid dose | 13.5 (-16.1-43.1) | 10.1 (-8.9-29.1) , P=0.314 | 17.3 (-6.5-41.2) , P=0.409 | 17.2 (-5.8-40.2) , P=0.136 | 13.7 (10.9-16.6) , P=0.975 | 12.2 (7.0-17.5) , P=0.835 |  |  |
|  |  | High dose | 8.8 (5.9-11.8) | 4.8 (1.8-7.8) , P=0.011 | 8.1 (5.6-10.6) , P=0.537 | 9.2 (7.3-11.1) , P=0.735 | 10.1 (7.5-12.7) , P=0.353 | 9.6 (7.1-12.1) , P=0.523 |  |  |
|  | Teres minor, lb, mean (95%CI) | Low dose | 8.8 (-10.8-28.5) | 1.1 (-3.5-5.7) , P=0.164 | 5.7 (-2.3-13.7) , P=0.43 | 5.7 (0.2-11.2) , P=0.519 | 7.9 (-2.9-18.7) , P=0.777 | 9.8 (-0.6-20.3) , P=0.734 |  |  |
|  |  | Mid dose | 9.3 (-3.4-22.0) | 3.6 (-7.6-14.7) , P=0.014 | 13.0 (-4.1-30.1) , P=0.162 | 14.0 (-1.5-29.5) , P=0.065 | 11.4 (8.4-11.4) , P=0.473 | 15.9 (8.8-23.1) , P=0.188 |  |  |
|  |  | High dose | 6.9 (3.6-10.3) | 1.2 (-0.0-2.5) , P=0.002 | 5.9 (4.1-7.8) , P=0.432 | 7.6 (6.2-9.0) , P=0.621 | 9.6 (7.4-11.7) , P=0.002 | 10.7 (7.8-13.6) , P=0.003 |  |  |
|  | SPADI, mean (95%CI) | Low dose | 44.1 (14.7-73.5) | 39.5 (19.2-59.7) , P=0.720 | 25.1 (-7.7-57.9) , P=0.305 | 18.5 (1.3-35.7) , P=0.09 | 10.0 (-9.1-29.1) , P=0.014 | 6.4 (1.6-11.2) , P=0.04 |  |  |
|  |  | Mid dose | 63.8 (42.8-84.9) | 49.2 (8.6-89.9) , P=0.187 | 25.6 (-17.8-69.1) , P=0.062 | 12.8 (-9.8-35.5) , P=0.020 | 8.3 (-3.6-20.1) , P<0.001 | 5.4 (-2.8-13.6) , P<0.001 |  |  |
|  |  | High dose | 75.4 (69.4-81.3) | 58.8 (52.3-65.2) , P<0.001 | 41.7 (30.8-52.7) , P<0.001 | 18.1 (13.7-20.0) , P<0.001 | 14.5 (9.0-20.0) , P<0.001 | 7.4 (2.6-12.1) , P<0.001 |  |  |
|  | Constant , mean (95%CI) | Low dose | 60.8 (19.4-102.1) | 29.3 (14.2-44.5) , P=0.139 | 55.8 (30.9-80.6) , P=0.774 | 61.7 (47.9-75.5) , P=0.926 | 72.1 (54.8-89.5) , P=0.339 | 74.6 (63.6-85.5) , P=0.373 |  |  |
|  |  | Mid dose | 60.9 (33.7-88.2) | 46.6 (15.2-78.0) , P=0.014 | 72.5 (43.5-101.5) , P=0.083 | 77.1 (56.3-97.9) , P=0.014 | 80.5 (76.4-84.6) , P=0.013 | 84.4 (74.8-93.9) , P<0.015 |  |  |
|  |  | High dose | 55.7 (51.8-59.6) | 31.5 (24.0-39.0) , P<0.001 | 51.4 (42.5-60.2) , P=0.323 | 66.3 (60.8-71.9) , P=0.007 | 74.0 (70.0-78.0) , P<0.001 | 80.3 (76.5-84.0) , P<0.001 |  |  |
|  | ASES, mean (95%CI) | Low dose | 55.4 (39.7-71.1) | 48.1 (42.9-53.4) , P=0.122 | 60.2 (12.5-107.8) , P=0.751 | 74.3 (53.5-95.0) , P0.154 | 79.6 (47.6-111.6) , P=0.148 | 84.8 (80.6-89.0) , P<0.017 |  |  |
|  |  | Mid dose | 45.9 (26.4-65.5) | 53.9 (9.5-98.2) , P=0.598 | 73.9 (24.8-122.9) , P=0.183 | 87.4 (55.6-119.2) , P=0.72 | 93.9 (83.3-104.4) , P<0.001 | 94.3 (85.9-102.7) , P<0.001 |  |  |
|  |  | High dose | 32.0 (26.8-37.2) | 31.5 (24.4, 38.5) , P=0.898 | 52.3 (42.7, 61.8) , P<0.001 | 76.6 (71.6, 81.6) , P<0.001 | 83.9 (78.3, 89.5) , P<0.001 | 89.4 (83.9, 95.0) , P<0.001 |  |  |
|  | UCLA, mean (95%CI) | Low dose | 18.7 (10.7-26.7) | 20.0 (11.0-29.0) , P=0.667 | 20.0 (-1.2-41.2) , P=0.827 | 22.7 (5.8-39.6) , P=0.543 | 25.3 (8.4-42.2) , P=0.362 | 31.3 (21.9-40.7) , P=0.078 |  |  |
|  |  | Mid dose | 18.3 (9.6-27.1) | 21.0 (6.1-35.9) , P=490 | 29.0 (19.1-38.9) , P=0.045 | 29.3 (22.2-36.5) , P=0.093 | 32.0 (29.3-34.8) , P<0.001 | 32.8 (29.0-36.5) , P<0.001 |  |  |
|  |  | High dose | 15.3 (14.2-16.4) | 17.3 (14.3-20.3) , P=0.266 | 20.0 (16.6-23.4) , P=0.017 | 28.4 (26.4-30.4) , P<0.001 | 30.3 (28.4-32.2) , P<0.001 | 32.2 (30.6-33.7) , P<0.001 |  |  |
|  | SST, mean (95%CI) | Low dose | 7.3 (3.5-11.1) | 1.0 (4.0-4.0) , P=0.063 | 5.2 (-2.3 - 12.9) , P=0.184 | 6.7 (2.9-10.5) , P=0.529 | 7.7 (6.2-9.1) , P=0.808 | 8.7 (3.5-13.8) , P=0.057 |  |  |
|  |  | Mid dose | 9.0 (4.7-13.3) | 8.0 (1.4-14.6) , P=0.225 | 10.3 (5.2-15.5) , P=0.57 | 11.0 (8.5-13.5) , P=0.074 | 11.4 (10.6-12.2) , P=0.118 | 10.9 (8.9-12.9) , P=0.203 |  |  |
|  |  | High dose | 4.4 (3.3-5.4) | 2.0 (0.8-3.1) , P<0.007 | 5.9 (4.2-7.6) , P=0.141 | 8.2 (7.1-9.4) , P<0.001 | 9.7 (8.6-10.9) , P<0.001 | 10.8 (10.0-11.6) , P<0.001 |  |  |
|  | DASH, mean (95%CI) | Low dose | 33.9 (2.2-65.6) | 46.1 (41.8-50.4) , P=0.218 | 33.9 (-20.5-88.3) , P=1.00 | 29.4 (-3.7-62.6) , P=0.784 | 17.5 (-9.6-44.6) , P=0.344 | 12.2 (5.6,-18.9) , P=0.083 |  |  |
|  |  | Mid dose | 40.0 (-6.7-86.7) | 39.7 (-7.7-87.1) , P=0.985 | 23.9 (-22.2-70.0) , P=0.281 | 11.7 (-5.3-28.6) , P=0.115 | 2.0 (-3.3-17.4) , P=0.008 | 5.2 (-2.4-12.8) , P=0.002 |  |  |
|  |  | High dose | 55.3 (47.2-63.4) | 62.3 (53.7-70.9) , P=0.181 | 38.6 (27.3-49.8) , P=0.002 | 22.0 (16.4-27.6) , P<0.001 | 17.1 (10.8-23.4) , P<0.001 | 9.1 (4.2-14.0) , P<0.001 |  |  |
|  | Overall function | Low dose | 40.0 (-3.0-83.0) | 33.3 (-4.61-71.3) , P=0.635 | 43.3 (29.0-57.7) , P=0.742 | 60.0 (35.2-84.8) , P=0.321 | 60.0 (-5.7-125.7) , P=0.438 | 83.3 (69.0-97.7) , P=0.083 |  |  |
|  |  | Mid dose | 36.7 (22.3-51.0) | 40.0 (-3.0-83.0) , P=0.808 | 66.7 (41.5-129.2) , P=0.225 | 73.3 (21.6-125.0) , P=0.128 | 89.3 (83.1-95.6) , P<0.001 | 93.7 (85.5-101.9) , P<0.001 |  |  |
|  |  | High dose | 37.7 (26.3-49.0) | 42.9 (31.8-54.1) , P=0.482 | 59.0 (51.1-66.9) , P<0.001 | 73.8 (66.6-81.1) , P<0.001 | 80.5 (73.7-87.3) , P<0.001 | 88.5 (84.6-92.5) , P<0.001 |  |  |
|  | Overall satisfaction | Low dose | - | 66.7 (28.7-104.6) | 56.7 (5.0-108.4) | 76.7 (62.3-91.0) | 76.7 (19.3-134.0) | 93.3 (79.0-107.7) |  |  |
|  |  | Mid dose | - | 53.3 (-58.7-165.3) | 80.0 (30.3-129.7) | 80.0 (14.3-145.7) | 90.3 (70.2-110.5) | 94.3 (84.6-104.1) |  |  |
|  |  | High dose | - | 57.2 (47.0-67.5) | 60.7 (53.2-68.2) | 82.0 (76.9-87.1) | 83.5 (76.7-90.4) | 93.4 (89.1-97.7) |  |  |
|  | failure: | Low dose |  |  |  |  |  |  | There was no failure of an injection of AD MSCs during 2 years of follow-up. |  |
|  |  | Mid dose |  |  |  |  |  |  |  |  |
|  |  | High dose |  |  |  |  |  |  |  |  |
|  | Articular, mm2%, mean (95%CI) | Low dose | 0 | 0 | 0 | 0 | 0 | 0 |  |  |
|  |  | Mid dose | 0 | 0 | 8.5 (-28.2-45.3) , P=0.423 | 0 | 0 | 0 |  |  |
|  |  | High dose | 41.8 (-15.7-99.4) | 32.9 (-49.8-115.5) , P=0.569 | 28.5 (1.9-55.0) , P=0.571 | 23.6 (-4.7-51.9) , P=0.506 | 13.5 (-7.6-34.5) , P=0.223 | 6.6 (-6.0-19.1) , P=0.195 |  |  |
|  | Bursal, mm2%, mean (95%CI) | Low dose | 62.0 (-152.3-276.4) | 56.3 (-141.6-254.2) , P=0.949 | 55.1 (-144.5-254.8) , P=0.941 | 59.8 (-121.2-240.8) , P=0.979 | 56.7 (-111.1-224.6) , P=0.948 | 11.1 (-25.8-48.0) , P0.346 |  |  |
|  |  | Mid dose | 53.6 (-55.4-162.6) | 41.9 (-75.0-158.8) , P=0.457 | 9.0 (-10.9-29.0) , P=0.166 | 16.9 (0.7-33.1) , P=0.321 | 30.3 (-9.7-70.2) , P=0.483 | 0 , P=0.257 |  |  |
|  |  | High dose | 27.7 (-0.9-54.5) | 17.1 (1.1-33.1) , P=0.349 | 3.4 (0.3-6.4) , P=0.019 | 1.0 (-1.1-5.2) , P=0.12 | 0.9 (-1.1-2.9) , P=0.018 | 0 , P=0.014 |  |  |
|  | Intratendinous, mm2%, mean (95%CI) | Low dose | 19.7 (-64.9-104.2) | 19.4 (-64.1-102.9) , P=0.423 | 14.7 (-48.7-78.2) , P=0.423 | 5.7 (-19.0-30.5) , P=0.423 | 5.7 (-19.0-30.5) , P=0.423 | 6.6 (-9.7-22.9) , P=0.513 |  |  |
|  |  | Mid dose | 0 | 0 | 0 | 0 | 1.6 (-13.1-16.4) , P=0.862 | 1.0 (-6.9-8.9) , P=0.808 |  |  |
|  |  | High dose | 4.8 (-4.7-14.4) | 7.4 (-6.0, 20.9) , P=0.725 | 9.5 (-3.1, 22.0) , P=0.544 | 15.3 (2.1, 28.4) , P=0.144 | 8.8 (-2.0, 19.6) , P=0.574 | 7.8 (-2.0, 17.5) , P=0.657 |  |  |
| L. N. Muench 2020 | forward elevation | Arthroscopic rotator cuff repair + BMC + PRP + subacromial bursa (N=16) | 130.3 ±46.0 |  |  |  | 174.0 ±9.9 , P=0.001 |  |  | This study showed that arthroscopic rotator cuff repair augmented with BMC improves patient function |
|  | abduction |  | 117.5 ±51.6 |  |  |  | 164.3 ±30.5 , P=0.001 |  |  |  |
|  | external rotation |  | 40.9 ±19.9 |  |  |  | 47.3 ±10.3 , P=0.162 |  |  |  |
|  | ASES |  | 45.8 ±22.5 |  |  |  | 88.5 ±14.6 , P=0.001 |  |  |  |
|  | SST |  | 4.3 ±3.2 |  |  |  | 10.4 ±1.6 , P=0.002 |  |  |  |
|  | Constant |  | 44.3 ±18.2 |  |  |  | 83.6 ±17.5 , P=0.001 |  |  |  |
|  | SANE |  | 13.3 ±10.7 |  |  |  | 86.3 ±17.5 , P=0.001 |  |  |  |
|  | Pain score |  | 5.0 ±2.8 |  |  |  | 1.1 ±1.6 , P=0.001 |  |  |  |
| **Elbow** | | | | | | | | | | |
| D. Connell 2009 | PRTEE scale | Elbow CEO tendinosis (tennis elbow, N=12) | 78 (IQR 71–88) | 47 (17.5–80) (p<0.05) | 35 (0–42) (p=0.01) | 12 (0–25) (p=0.003) |  |  | No adverse effect | Tendon like cells have a therapeutic effect on refractory CEO tendinosis |
|  | Ultrasound |  | Thickness(mm) = 4.35 |  |  | Thickness(mm) = 4.20 (p=0.015) |  |  |  |  |
|  |  |  | Hypoechogenicity = 7 |  |  | Hypoechogenicity = 3 (p=0.002) |  |  |  |  |
|  |  |  | Neovascularity = 3 |  |  | Neovascularity = 1 (p=0.008) |  |  |  |  |
|  |  |  | Tear (mm) = 5 |  |  | Tear (mm) = 2 (p=0.002) |  |  |  |  |
| A. Singh 2014 | PRTEE scale | Elbow CEO tendinosis BMC injeceted group (N=26) | 72.8 ± 6.97 | 24.46 ± 4.58  (P<0.0001) | 14.86 ± 3.48  (P<0.0001) |  |  |  |  | significant improvement of pain relief and recovery from the disease following a single injection of BMC |
| Allan Wang 2015 | VAS (maximum pain) | Tenocytes injection (N=16) | 5.94 ± 2.24 | 2.3 , P<0.001 | 1.7 , P<0.001 | 1.1 , P<0.001 | 0.7 , P<0.001 | 1.21 ± 1.24, P<0.001 | At final follow-up, 93% (n = 14) of patients were either highly satisfied or satisfied with their ATI treatment outcome. | Tenocyte injection for Lateral Epicondylitis treatment showed an acceptable results in function and structure improvement. |
|  | QuickDASH score |  | 45.88 ± 15.24 | 17.4 , P<0.001 | 18.5 , P<0.001 | 9.6 , P<0.001 | 2.88 ± 0.72 , P<0.001 | 6.61 ± 7.48, P<0.001 |  |  |
|  | Grip strength (kg) |  | 19.85 ± 11.24 | 25.1 , P<0.001 | 28.5 , P<0.001 | 37.4 , P<0.001 | 37.4 , P<0.001 | 46.60 ± 13.84, P<0.001 |  |  |
|  | UEFS |  | 31.73 ± 15 |  |  |  |  | 9.20 ± 0.39, P<0.001 |  |  |
|  | MRI (total) |  | 4.31 ± 1.12 |  |  |  |  | 2.87 ± 0.76 |  |  |
| Lee, S. Y 2015 | VAS (pain on motion) | AD-MSC low dose (N=6) | 67.3 | 50 , P<0.01 | 38 , P<0.01 | 16.6 , P<0.01 | 11 , P<0.01 |  |  | Allogenic AD-MSC injection for lateral epicondylosis treatment is safe and effective. |
|  |  | AD-MSC high dose (N=6) | 61.6 | 31 , P<0.01 | 22.3 , P<0.01 | 13 , P<0.01 | 17.3 , P<0.01 |  |  |  |
|  | MEPI | AD-MSC low dose | 57.5 , P<0.01 | 82.3 , P<0.01 | 86.8 , P<0.01 | 91.8 , P<0.01 | 90.6 , P<0.01 |  |  |  |
|  |  | AD-MSC high dose | 71.3 | 88.8 , P<0.01 | 88.4 , P<0.01 | 49.3 , P<0.01 | 88.1 , P<0.01 |  |  |  |
|  | Tendon defect area (longitudinal, mm2) by US | AD-MSC low dose | 4.8 | 5 | 4 | 1.7, P<0.01 | 2.3, P<0.01 |  |  |  |
|  |  | AD-MSC high dose | 7.8 | 5.3 | 5.2 | 2.8, P<0.01 | 3.7, P<0.01 |  |  |  |
|  | Tendon defect area (transverse, mm2) by US | AD-MSC low dose | 8.1 | 6.5 | 5.1 | 2.1, P<0.01 | 3, P<0.05 |  |  |  |
|  |  | AD-MSC high dose | 7.3 | 7.1 | 7.4 | 3.6, P<0.01 | 5.2, P<0.05 |  |  |  |
| Miguel Khoury 2021 | VAS | AD-MSC (N=18), no controls | 6.28 ± 1.65 |  | 1.05 ± 0.43 , P*<0.001 |  |  |  |  | Recalcitrant LET in tennis players showed clinical improvement and anatomical repair after autologous ASCs injection. |
|  | QuickDASH-Compulsory |  | 51.38 ± 12.02 |  | 12.33 ± 4.66 , P*<0.001 |  |  |  |  |  |
|  | QuickDASH-Sport |  | 56.94 ± 15.44 |  | 8.68 ± 8.86 P*<0.001 |  |  |  |  |  |
|  | Total MRI score |  | 4.22 ± 0.26 |  | 2.22 ± 0.10 , P*<0.001 |  |  |  |  |  |
| **Achilles** |  |  |  |  |  |  |  |  |  |  |
| Kristin Tate-Oliver 2013 | VAS | AD-tSVF +HD-PRP patient #1 | 9 |  | 1 |  |  | 0 |  | Use of AD-SVF and HD-PRP and/or BMAC is safe and a good option for Achilles tendonitis management without surgery |
|  |  | AD-tSVF +HD-PRP patient #2 | 9 |  |  |  |  | 0 |  |  |
|  |  | AD-tSVF +HD-PRP + BMC | 10 |  |  |  |  | 0 |  |  |
| Stein, B. E 2015 | Mean difference in calf circumference | BMC injection group (N=27) |  |  |  |  |  | −0.5 (−2.0 - 3.0) | Superficial wound dehiscence (N=1) , ipsilateral knee pain (N=1) | patient with Achilles tendon repairs treated with BMAC injection shows a great functional rate of return to sport, rehabilitation progress, and single-limb heel raise outcomes. |
|  | Time to walking without boot |  |  |  |  |  |  | 1.8 (0.8 - 3.5) |  |  |
|  | Time to light activity |  |  |  |  |  |  | 3.4 (1.2 - 10.0) |  |  |
|  | Returned to sport |  |  |  |  |  |  | 92 % (25 / 27) |  |  |
|  | Time to return to sport |  |  |  |  |  |  | 5.9 ( 3.0 - 9.0) |  |  |
|  | Mean ATRS |  |  |  |  |  |  | 91 (72 - 100) |  |  |
|  | ROM |  |  |  |  |  |  | All patients achieved acceptable ROM and were able to perform a single-limb heel raise at final follow-up successfully | |  |
| Usuelli, F. G 2017 | VAS | PRP group(N= 23) |  |  | 6.3±1.2 |  |  |  | After 180 days from treatment, the lesion area was not significantly reduced in either PRP or SVF patients (8.67±2.10 and 10.46±3.37 mm2) | We can use both PRP and SVF to treat recalcitrant Achilles tendinopathy, and it's safe and effective. However, we can obtain results faster in SVF treatment. |
|  |  | SVF group(N=21) |  |  | 6.5±1.6 , P*>0.05 |  |  |  |  |  |
|  | VISA | PRP group |  |  | 46.5±23.6 |  |  |  |  |  |
|  |  | SVF group |  |  | 41.6±13.6 , P*>0.05 |  |  |  |  |  |
|  | AOFAS | PRP group |  |  | 63.2±17.7 |  |  |  |  |  |
|  |  | SVF group |  |  | 63.4±20.1 , P*>0.05 |  |  |  |  |  |
|  | SF-36 P | PRP group |  |  | 38.5±7.9 |  |  |  |  |  |
|  |  | SVF group |  |  | 42.2±5.5 , P*>0.05 |  |  |  |  |  |
|  | SF-36M | PRP group |  |  | 51.21±8 |  |  |  |  |  |
|  |  | SVF group |  |  | 48.7±5.7 , P*>0.05 |  |  |  |  |  |
|  | The mean preoperative lesion area assessed MRI and US assessment | PRP group |  |  | 8.93±2.10 |  |  |  |  |  |
|  |  | SVF group |  |  | 10.70±3.38 |  |  |  |  |  |
| **Patellar** | | | | | | | | | | |
| A. W. Clarke 2011 | VISA | plasma-only injection (N=27) | 50±18 |  |  | 70±14 |  |  |  | Patellar tendinopathy treated with skin-derived tendon-like cells can be safely treated in the short term, with significantly better outcome than that achieved with plasma alone. |
|  |  | Tenocyte-like Cell + plasma (N=33) | 44±15 |  |  | 75±17 , P*=-0.006 |  |  |  |  |
|  | US thickness | plasma-only injection | 8.513± 2.282 |  |  | 7.517± 1.822, P= 0.1386 |  |  |  |  |
|  |  | Tenocyte-like Cell + plasma | 9.146± 1.666 |  |  | 7.913± 1.368, P= 0.0072 |  |  |  |  |
|  | US hypoechogenisity | plasma-only injection | 6.261± 1.959 |  |  | 3.828± 1.79, P=0.0002 |  |  |  |  |
|  |  | Tenocyte-like Cell + plasma | 6.52± 1.584 |  |  | 4.13± 2.302, P=0.0001 |  |  |  |  |
|  | US Tear size | plasma-only injection | 8.613±4.977 |  |  | 1.822± 1.686, P=0.0072 |  |  |  |  |
|  |  | Tenocyte-like Cell + plasma | 10.47± 7.146 |  |  | 2.414± 2.194, P<0.0001 |  |  |  |  |
|  | US neovascularity | plasma-only injection | 2.304± 2.754 |  |  | 2.353± 2.149, P=0.6508 |  |  |  |  |
|  |  | Tenocyte-like Cell + plasma | 2.28± 2.227 |  |  | 2.696± 2.687, P=0.7248 |  |  |  |  |
| C. Pascual-Garrido 2012 | Ultrasound grading | BM-MNC group (N=8, No control group) | Grade 2/3 (N=8) |  |  | Grade 1 (N=8) |  |  | Patients intention to repeat the same treatment: yes= 7 (completely satisfied), No complications | BM-MNC therapy improves chronic patellar tendinopathy after nonoperative treatment’s failure |
|  | Lysholm |  | 33 |  |  |  |  | 53 (P=0.104) |  |  |
|  | Tegner |  | 2 |  |  |  |  | 8 (P=0.006) |  |  |
|  | IKDC |  | 36 |  |  |  |  | 69 (P=0.047) |  |  |
|  | KOOS |  | Pain=47 |  |  |  |  | Pain=63 (P=0.2399) |  |  |
|  |  |  | Symptoms =44 |  |  |  |  | Symptoms =71 (P=0.0086) |  |  |
|  |  |  | ADL=63 |  |  |  |  | ADL=90 (P=0.0246) |  |  |
|  |  |  | Sport =24 |  |  |  |  | Sport =63 (P=0.0078) |  |  |
|  |  |  | QOL=50 |  |  |  |  | QOL=71 (p=0.0825) |  |  |
|  | SF12 |  | Mental =52 |  |  |  |  | Mental =57 (p=0.5589) |  |  |
|  |  |  | Physical =41 |  |  |  |  | Physical =44 (p=0.438) |  |  |
| Gil Rodas 2021 | VAS mean | Lp-PRP group (N=10) | 3.10±1.20 |  |  | 1.13±1.25 , P= 0.008 |  |  |  | This study confirmed that treatment with BM-MSC or Lp-PRP could reduce the pain; however, BM-MSC is more effective |
|  |  | MSC group (N=10) | 4.23±2.13 |  |  | 2.52±2.37 , P= 0.062 , P*>0.05 |  |  |  |  |
|  | VAS in motion | Lp-PRP group | 7.03±1.42 |  |  | 1.94±1.24 , , P< 0.001 |  |  |  |  |
|  |  | MSC group | 6.91±1.11 |  |  | 3.06±2.89 , P= 0.0049 , P*>0.05 |  |  |  |  |
|  | VISA | Lp-PRP group | 47.00±9.83 |  |  | 72.90±17.34 , P< 0.001 |  |  |  |  |
|  |  | MSC group | 42.30±16.29 |  |  | 66±26.66 , P<0.001 , P*>0.05 |  |  |  |  |
|  | UTC algorithms echo types I | Lp-PRP group | 51.70±16.07 |  |  | 47.89±6.39 |  |  |  |  |
|  |  | MSC group | 53.10±13.45 |  |  | 53.10±13.45 , P*=0.042 |  |  |  |  |
|  | UTC algorithms echo types II | Lp-PRP group | 37.13±9.65 |  |  | 36.37±6.07 |  |  |  |  |
|  |  | MSC group | 35.06±8.83 |  |  | 35.02±6.98 , P*=0.09 |  |  |  |  |
|  | UTC algorithms echo types III | Lp-PRP group | 7.87±7.52 |  |  | 9.19±4.64 |  |  |  |  |
|  |  | MSC group | 8.01±8.24 |  |  | 4.35±7.78 , P*=0.009 |  |  |  |  |
|  | UTC algorithms echo types IV | Lp-PRP group | 3.37±3.35 |  |  | 4.82±3.42 |  |  |  |  |
|  |  | MSC group | 3.91±3.89 |  |  | 1.80±3.38 , P*=0.016 |  |  |  |  |
|  | US Area, mm2 | Lp-PRP group | 8.67±10.47 |  |  | 9.64±5.28 , P= 0.787 |  |  |  |  |
|  |  | MSC group | 15.06±15.56 |  |  | 3.38±3.49 , P= 0.0195 , P*=0.016 |  |  |  |  |
|  | US Volume, mm3 | Lp-PRP group | 40.04±46.48 |  |  | 31.68±23.49 , P= 0.945 |  |  |  |  |
|  |  | MSC group | 72.91±117.93 |  |  | 9.15±16.79 , P= 0.0156 , P*=0.037 |  |  |  |  |
|  | MRI Size of the lesion on T2-weighted, fat-saturated coronal image | Lp-PRP group | 14.00±8.43 |  |  | 18.00±4.22 , P= 0.25 |  |  |  |  |
|  |  | MSC group | 15.00±7.07 |  |  | 19.00±8.76 , P= 0.168 , P*=0.72 |  |  |  |  |
|  | MRI Passage of fibers/fascicles on T2-weighted, fat-saturated sagittal and coronal images | Lp-PRP group | 23.30±5.66 |  |  | 20.30±8.31 , P= 0.224 |  |  |  |  |
|  |  | MSC group | 14.30±5.17 |  |  | 24.60±3.17 , P<0.0001 , P*<0.001 |  |  |  |  |
|  | MRI Hypersignal in T2-weighted on spin-echo T2-weighted coronal image | Lp-PRP group | 5.40±2.22 |  |  | 6.00±1.63 , P= 0.536 |  |  |  |  |
|  |  | MSC group | 4.00±1.25 |  |  | 6.20±1.23 , P<0.0001 , P*=0.0106 |  |  |  |  |
|  | MRI Hoffa fat edema on T2-weighted, fat-saturated sagittal and axial images | Lp-PRP group | 6.50±2.07 |  |  | 6.40±1.96, P=0.823 |  |  |  |  |
|  |  | MSC group | 3.80±2.30 |  |  | 5.00±2.54, P=0.154 , P*=0.386 |  |  |  |  |
|  | MRI Lower patellar bone edema on T2-weighted, fat-saturated sagittal and axial images | Lp-PRP group | 7.50±1.90 |  |  | 7.00±2.21, P=0.40 |  |  |  |  |
|  |  | MSC group | 5.40±2.17 |  |  | 5.10±2.13, P=0.67 , P*=614 |  |  |  |  |
|  | All MRI items | Lp-PRP group | 56.70±7.44 |  |  | 57.70±14.02, P=0.824 |  |  |  |  |
|  |  | MSC group | 42.50±8.05 |  |  | 59.90±11.51 , P<0.001 , P*0.004 |  |  |  |  |
| **Gluteal** | | | | | | | | | | |
| D. A. V. Rosário 2021 | VAS (Median (IQR)) | Corticosteroid group (N=25) | 6.0 (4.0 – 7.5) | 3.0 (2.0 – 5.0) |  | 4.0 (2.5 – 6.0) , P=0.001 |  |  |  | This study approve that BMAC is safe and effective to treat gluteal tendinopathy |
|  |  | BMC group (N=15) | 6.0 (5.0 – 7.0) | 3.0 (2.0 – 4.0) |  | 2.0 (2.0 – 3.0) , P=0.001 , P*=0.001 |  |  |  |  |
|  | EuroQol 5D (Average±SD) | Corticosteroid group | 0.50±0.33 |  |  | 0.61±0.25 , , P=0.016 |  |  |  |  |
|  |  | BMC group | 0.52±0.22 |  |  | 0.68±0.18 , P=0.002 , P*=0.590 |  |  |  |  |
|  | Lequesne | Corticosteroid group | 8.0±3.6 |  |  | 6.8±3.3 , P=0.021 |  |  |  |  |
|  |  | BMC group | 8.7±1.9 |  |  | 4.7±2.1 , P=0.001 , P*<0.001 |  |  |  |  |
